# NAD^+^ Metabolism-Mediated SURF4-STING Axis Enhances T-Cell Anti-Tumor Effects in the Ovarian Cancer Microenvironment

**DOI:** 10.1038/s41419-025-07939-9

**Published:** 2025-08-23

**Authors:** Jiacheng Shen, Fangfang Xu, Tingwei Liu, Yingjun Ye, Shaohua Xu

**Affiliations:** Department of Gynecology, Shanghai Key Laboratory of Maternal Fetal Medicine, Shanghai Institute of Maternal-Fetal Medicine and Gynecologic Oncology, Shanghai First Maternity and Infant Hospital, School of Medicine, Tongji University, Shanghai, 200092 China

**Keywords:** Ovarian cancer, Cancer microenvironment

## Abstract

The anti-tumor function of T cells in the ovarian cancer (OC) microenvironment influences the prognosis of OC. Previous studies have indicated that metabolic competition among microenvironmental cells regulates the function of immune cells. Recent research has shown that NAD^+^ metabolism plays a significant role in modulating immune cell activity, and increasing NAD^+^ levels is a promising therapeutic strategy to enhance the effector functions of immune cells. However, the regulatory mechanisms of NAD^+^ metabolism on the anti-tumor function of T cells in the OC microenvironment remain unclear. This study found that exogenous supplementation of NAM to increase NAD^+^ levels in T cells significantly activates the endogenous p-STING axis and downstream interferon signaling within T cells, thereby enhancing T cell activation and anti-tumor effects. Concurrently, we discovered that elevated NAD^+^ levels promote the retention of STING on the Golgi apparatus. Mechanistically, we elucidated that the increase in NAD^+^ levels mediated by NAM downregulates the expression of SURF4 protein through ubiquitination and degradation, subsequently activating the p-STING axis in T cells. Furthermore, exogenous NAM supplementation can further enhance the activation of the T cell STING axis by PARP inhibitor (PARPi)-treated OC cells, and the combination of PARPi and NAM significantly augments the anti-tumor function of T cells, inhibiting the progression of OC. Our findings provide a molecular basis for the regulation of T cell anti-tumor function by NAD^+^, highlighting the potential strategy of targeting T cell metabolic reprogramming for the treatment of OC.

## Introduction

Ovarian cancer (OC) remains a leading cause of cancer-related mortality due to its highly aggressive nature and propensity for recurrence [1]. Although current first-line chemotherapy regimens have improved patient outcomes to some extent, the majority of patients eventually develop drug resistance [2, 3]. Furthermore, the low immune responsiveness of the OC microenvironment significantly impedes the efficacy of immunotherapies [4, 5]. The tumor immune microenvironment (TIME) of OC plays a pivotal role in tumor progression, immune evasion, and treatment resistance [6–8]. Despite existing research elucidating the complexity of OC TIME, our understanding of the regulatory factors governing the immune status in this microenvironment remains limited.

T cells represent a crucial cellular component of anti-tumor immunity. In the OC microenvironment, the prevalent immunosuppressive milieu often constrains the antitumor efficacy of T cells [9]. The functional activation of T cells is associated with the regulation of various endogenous signaling pathways. As a critical regulator of cancer immunity, the stimulator of interferon genes (STING) signaling pathway demonstrates a close relationship with cancer development [10]. Contrary to the prevailing view that this axis primarily bridges innate and adaptive immunity, increasing evidence suggests that adaptive immune cells can directly respond to STING engagement [11]. In recent years, the functional role of STING signaling in T cells has garnered increasing attention, particularly in regulating T cell survival, differentiation, and immune suppression. Research indicates that many STING signaling functions in T cells may be independent of type I interferon [12]. However, the role of endogenous STING signaling in T cell responses remains largely unexplored, and understanding the direct effects of STING pathway in T cells will facilitate the rational design of novel therapeutic strategies.

Metabolism plays a significant role in the tumor microenvironment (TME), where metabolic competition among different cellular components regulates immune cell function [13, 14]. As a crucial aspect of cellular activities, NAD^+^ metabolism has been suggested to play an important role in regulating immune cell activity [15, 16]. In this context, NAD^+^ availability becomes a limiting resource, and the competition for NAD^+^ between tumor cells and immune cells represents a key mechanism of tumor-mediated suppression of anti-tumor immunity. Immune cells, particularly T cells and macrophages, require NAD^+^ to support their activation, proliferation, and effector functions [17, 18]. Therefore, in-depth investigation of NAD^+^ metabolism in the OC microenvironment and its regulatory mechanisms on endogenous signaling pathways in T cells is of great significance for exploring approaches to enhance T cell anti-tumor activity.

This study reveals the existence of NAD^+^ level competition between tumor cells and T cells in the OC microenvironment. Low NAD^+^ levels in T cells suppress their anti-tumor function. Exogenous supplementation with the NAD^+^ precursor NAM can significantly enhance T cell anti-tumor effects, mediated through STING axis activation. We explored potential mechanisms of NAD^+^ level regulation of STING axis activity. Additionally, this study investigates the potential role of NAM supplementation in enhancing PARP inhibitor (PARPi)-mediated anti-tumor immunity by increasing T cell NAD^+^ levels, providing a theoretical foundation for developing novel therapeutic approaches.

## Materials and Methods

### OC single-cell data clustering and subpopulation identification

**Fig. 1:**
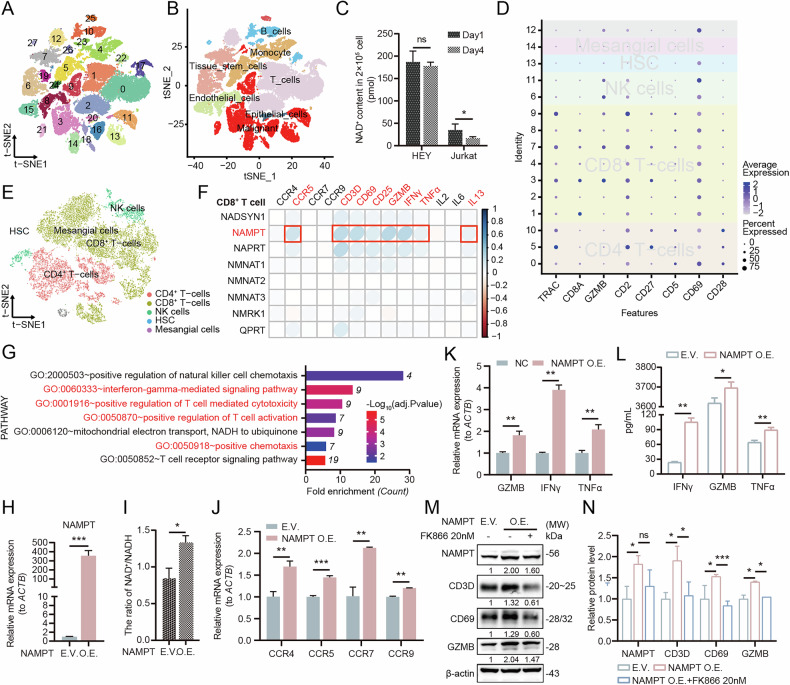
NAMPT-mediated NAD^+^ biosynthesis regulates T cell function in OC microenvironment. **A**, **B** Dimensionality reduction, clustering, and cell annotation of single-cell data from OC (GSE217517). **A** A total of 28 cell clusters were identified, with each color representing a distinct cell cluster. **B** Discrimination between malignant and non-malignant cells, along with cell annotation. Six cell types were identified among the non-malignant cells, with each color denoting a specific cell type. **C** The NAD^+^ content in T cells was found to be abnormally reduced during co-culture with OC cells. **D** Marker genes are used for the subtype identification of T cells. **E** T cell subsets can be clustered into five distinct cell types: HSC, NK cells, Mesangial cells, CD8^+^ T-cells, CD4^+^ T-cells. **F** The expression of the NAD^+^ synthesis enzyme NAMPT is closely correlated with the expression of T cell activity genes. **G** NAMPT shows a positive correlation with T cell anti-tumor signaling. **H** Transcriptional level detection of NAMPT overexpression in T cells. **I** Overexpression of NAMPT increases the NAD^+^ levels within T cells. **J** NAMPT promotes the expression of T cell chemotaxis receptors. **K**, **L** NAMPT enhances the expression of T cell anti-tumor factors IFNγ, GZMB and TNFα. **M**, **N** NAMPT upregulates the expression of T cell infiltration and activation markers (CD3D, CD69, and GZMB), while FK866 can effectively suppress T cell reactivation.

OC single-cell dataset GSE217517 was downloaded from GEO (https://www.ncbi.nlm.nih.gov/geo/) for investigating the intra-tumoral microenvironment [19]. The ‘Seurat’ package was used for data normalization, dimensionality reduction, and clustering of single-cell data, with the RunTSNE function employed for dimensionality reduction and data visualization. Consistent with the methods we used in our previous research, we extracted malignant epithelial cells from the single-cell data using marker proteins WT1, PAX8, and MUC16, labeled as “Malignant”, while the remaining cells were labeled as “Non-malignant” (Fig. 1B, Fig. S1D) [20].

The ‘SingleR’ package was utilized to identify cell types within the single-cell data. For the broad classification of cell types, the cell type annotation method from the HumanPrimaryCellAtlasData (HPCA) database within the ‘celldex’ package was employed. To further delineate functional subsets of T cell populations, the BlueprintEncodeData database from the ‘celldex’ package was applied. Visualization of the proportions of various cellular components across all samples was conducted using the ggplot function.

### Differential expression genes (DEGs), functional enrichment, and protein-protein interaction analysis

The ‘limma’ package was used for differential gene screening, with DEGs identified based on filtering criteria: adj.p.value < 0.05 and |Fold Change | > 1.5.

The ‘ClusterProfiler’ package was used to identify significantly enriched Gene Ontology (GO) terms and pathways from the Kyoto Encyclopedia of Genes and Genomes (KEGG), Reactome, and WIKIPATHWAY databases. Gene Set Enrichment Analysis (GSEA) was also performed using the ‘ClusterProfiler’ package, with all results visualized using gseaplot2.

Predicted protein-protein interactions (PPI) were generated using the Genemania website (https://genemania.org/), and interaction networks were reconstructed using Cytoscape.

### Cell culture and drug treatment

The human leukemia T lymphocyte Jurkat (E6-1 clone, JE-6) cells, human OC cell line SKOV3, human OC cell line HEY, human renal epithelial cell line 293 T cells (HEK 293 T), and mouse ovarian epithelial carcinoma ID8 cells used in this study were all purchased from Procell Life Science & Technology Co., Ltd. and Guangzhou Saiku Biotechnology Co., Ltd. JE-6 cells and SKOV3 cells were cultured in RPMI-1640 medium (KGL1503, KeyGEN) supplemented with 10% fetal bovine serum (FBS; FBS03, SinoBiological) and 1% penicillin-streptomycin (P/S; G4003, Servicebio). HEY cells, HEK 293 T cells, and ID8 cells were maintained in DMEM (high glucose; KGL1211, KeyGEN) containing 10% FBS and 1% P/S. All cell lines were cultured at 37 °C in a humidified atmosphere with 5% CO_2_.

The small molecules used for cell treatment included: Daporinad (FK866, HY-50876, MCE), Nicotinamide (NAM, 4106, R&D), NMN (HY-F0004, MCE), NR (HY-123033A, MCE), H-151 (1 μM; HY-112693, MCE), ionomycin (500 ng/mL; HY-13434, MCE), PMA (20 ng/mL; V1171, Promega), Olaparib (HY-10162, MCE), RU.521 (10 μM; M9447, AbMole), MG-132 (10 μM; HY-13259, MCE), and Cycloheximide (CHX; 10 μg/mL; M4879, AbMole). FK866, H-151, Olaparib, PMA, RU.521, MG-132, and CHX were dissolved and diluted in DMSO (D8371, Solarbio). NAM, NMN, and NR were prepared in sterile water, while ionomycin was dissolved and diluted in absolute ethanol. JE-6 T cell activation was achieved by adding ionomycin and PMA 4–6 h prior to cell collection.

For the preparation of T cell supernatant used to treat OC cells or humanized OC organoids, to avoid direct effects of small molecule drugs on OC cells and organoids, the culture medium was replaced with fresh medium 24 h after small molecule treatment of T cells, followed by supernatant collection for subsequent treatment of OC cells or organoids.

### Extraction and culture of patient-derived ovarian cancer organoids (PDOCO)

In this study, the human biological samples involved were approved by the hospital ethics committee. All samples were handled and processed according to the committee’s standards. Detailed information on the clinical OC samples can be found in Table S1. OC tissues obtained from clinical sources must be processed within 24 h to ensure organoid viability. The OC tissue is washed and minced, then digested using AdDF12 containing DNAase (10 U/mL; DN25, Sigma), Y-27632 (5 μM; C629401, bioGenous), and Collagenase I (500 U/mL; C0130, Sigma). After digestion, the mixture is neutralized and filtered through a 100 μm cell strainer. Red blood cells are removed using red blood cell lysis buffer (B541001, Sangon), and the remaining cells are resuspended in an appropriate amount of BME matrix gel (3533-010-02, R&D). The cell-matrix mixture is plated, and the plate is inverted and placed in a 37 °C, 5% CO_2_ incubator for 30 minutes to allow the gel droplets to solidify. After solidification, PDOCO complete medium OCOM (ovarian cancer organoid medium, Table S2, prepared according to the methods described in studies by Kopper O et al. and Maenhoudt N et al. [21, 22]) is added, and the organoids are cultured in a 37 °C, 5% CO_2_ incubator. The OCOM medium is replaced with fresh medium every 3 days.

During PDOCO passaging, TrypLE (12604021, Gibco) is used for digestion. For cryopreservation of PDOCO, a self-prepared cryopreservation solution (AdDF12:FBS:DMSO = 1:8:1) is used. The samples are placed in a programmed freezing box and stored at –80°C, then transferred to liquid nitrogen for long-term storage.

### Cell transfection

For NAMPT or SURF4 overexpression, the pLVX-Puro-Flag-NAMPT and pLenti-CMV-SURF4-HA-EF1-GFP-Puro lentiviral vector and empty vector were obtained from PPL (Public Protein/Plasmid Library). For SURF4 downregulation, pPLK/GFP-Puro-SURF4 shRNA (1#shRNA: GCTCTTTGCCATCAACGTATA, 2#shRNA: CCCATGCATGACTTCCTGAAA) and a vector containing a scrambled shRNA were purchased from PPL. With reference to a protocol described previously [23], lentiviruses were generated by triple transfection of the above plasmids and helper plasmids into HEK 293 T cells and were then purified from the cell culture medium using PEG8000 solution.

The virus solution was mixed with RPMI-1640 containing 10% FBS at a 3:2 ratio, and 7 × 10^5 ^T cells were resuspended in the mixture. Polybrene (H8761, Solarbio) was added at a concentration of 10 μg/mL, and the cells were centrifuged at 37 °C, 1000 G for 2 h. The culture plate was then placed in a 37 °C, 5% CO_2_ incubator. After 24 h, the culture medium was replaced with fresh RPMI-1640 containing 10% FBS and continued to be cultured. Starting from the third day after lentivirus infection, positive cells were screened with 1 μg/mL puromycin (ST551, Beyotime).

### Cell co-culture

Indirect Co-Culture: 1.5 mL of OC cells at a density of 1 × 10^5^ cells/mL were plated in a 6-well plate. After HEY cells adhered, 1 mL of T cells at a density of 6 × 10^5^ cells/mL was added to the co-culture insert. The co-culture was then placed in a 37 °C incubator, and NAD^+^ content was detected after 24 h and 96 h of co-culture. A small amount of culture medium was supplemented on the second day.

Direct Co-Culture: First, 4 × 10^5^ OC cells were plated in a well plate. After adherence, a cell suspension containing 2 × 10^6 ^T cells was slowly added. After 24 h of culture, the upper layer of T cells could be gently collected with a pipette, while the lower layer of OC cells was directly collected with RNA or protein lysis buffer.

### CCK-8 assay, colony formation, and chemotaxis assay

CCK-8 Assay: JE-6 cells were plated at a density of 3000 cells per well. SKOV3 and HEY cells were plated at a density of 1000 cells per well. During the assay, 10 μL of CCK-8 reagent (A311-02, Vazyme) was added to each well, followed by incubation at 37 °C in the dark for several hours. The OD values at 450 nm were measured at 0 h, 24 h, 48 h, 72 h, and 96 h.

Colony Formation: OC cells were plated at a density of 1000 cells per well in 6-well plates. After adherence, a 1:1 mixture of T cell supernatant and fresh culture medium was added, and the medium was replaced every two days. On day 12, the cells were fixed, stained with crystal violet (G1014, Servicebio), and photographed against a white background.

Chemotaxis Assay: T cells treated were resuspended at a density of 1 × 10^6^/mL in 100 μL fresh RPMI-1640 and added to the upper chamber of a 24-well co-culture plate. The lower chamber contained 500 μL of RPMI-1640 culture medium with 10% FBS. After 24 h of culture, the cells in the lower chamber were counted.

### Cell cycle and apoptosis assays

Cell cycle analysis was performed using the Cell Cycle and Apoptosis Assay Kit (C1052, Beyotime). Apoptosis was detected using the Annexin V-FITC/PI Apoptosis Detection Kit (40302ES, Yeasen Bio). All procedures were carried out according to the manufacturer’s instructions. The cell cycle of each sample was statistically analyzed using MFLT32 software, and the apoptosis results were analyzed using FlowJo software.

### Cytotoxicity assay

The treated JE-6 cells (E) were mixed with OC cells (T) Luciferase-GFP-SKOV3 or Luciferase-GFP-HEY at an effector-to-target ratio (E:T) of 9:1 and plated in opaque 96-well culture plates for co-culture for 24 h. The next day, 100 μL of ONE-Glo Luciferase Assay System reagent (E6110, Promega) was added to each well. After standing for 3 min to ensure complete cell lysis, the luminescence values were measured using a microplate reader.

### NAD^+^/NADH content detection

The NAD^+^/NADH content in cells was measured using the NAD^+^/NADH Detection Kit (WST-8 method) (S0175, Beyotime). All procedures were carried out according to the manufacturer’s instructions.

### RT-qPCR

Total RNA was extracted from tissues and cells using the RNA extraction solution (RK30129, Abclonal) and converted into cDNA using the ABScript Neo RT Master Mix (RK20433, Abclonal) according to the manufacturer’s instructions. RT-qPCR was performed using SYBR Green qPCR Master Mix (RK21219, Abclonal) with the reaction system and program according to the manufacturer’s instructions. RNA expression levels were normalized to β-actin, and ΔΔCT values were calculated for expression quantification. All primers were synthesized by Sangon Biotech (Table S3).

### Western blot

Total protein samples were extracted from OC cell lines and tissues using protein lysis (P0013, Beyotime) supplemented with 1% protease/phosphatase inhibitors (P001, NCM). Proteins (20 μg/lane) were separated using 8–12% SDS-PAGE and then transferred onto a PVDF membrane (IPVH00010, Millipore). The membrane was then blocked in 5% nonfat milk and incubated overnight with specific primary antibodies at 4°C. The primary antibodies include: anti-NAMPT (1:1000; CSB-PA015422EA01HU, Cusabio), anti-CD8 (1:1000; T40010, Abmart), anti-CD69 (1:1000; 10803-1-AP, Proteintech), anti-CD3D (1:1000; 16669-1-AP, Proteintech), anti-GZMB (1:1000; 16669-1-AP, Proteintech), anti-STING (1:5000; A21051, Abclonal), anti-pSTING (1:1000; AP1369, Abclonal), anti-IRF3 (1:1500; A19717, Abclonal), anti-pIRF3 (1:5000; AP1412, Abclonal), anti-SURF4 (1:1000; A16744, Abclonal), anti-Ubiquitin (1:1000; A19686, Abclonal), anti-DDDDK-Tag (1:20000; AE005, Abclonal), anti-HA (1:2000, AE008, Abclonal), anti-β-actin (1:10000; AC026, Abclonal), anti-HRP-conjugated GAPDH (1:10000; HRP-60004, Proteintech), and anti-α-tubulin (1:3000; GB11200, Servicebio). After incubation with the corresponding secondary antibodies, bands were detected using ECL chemiluminescence substrate (SQ203, Epizyme). The gray values of the Western blot were normalized to β-actin.

### Immunoprecipitation

The total protein extraction method is the same as described in Section “Western Blot”. An appropriate amount of magnetic beads (RM02915, Abclonal) and corresponding antibodies are added according to the protein concentration. The mixture is then placed on a rotating mixer and incubated at 4 °C overnight for crosslinking. The next day, the complexes are washed three times with IP wash buffer (ddH_2_O contains 10% NP-40 (N8030, Solarbio), 5 M NaCl (A501218, Sangon), 1 M Tris-HCl (ST775, Beyotime), and 10% SDS (G2055, Servicebio)), and the complexes are resuspended in protein loading buffer. The antigens are eluted by heating at 100 °C for 10 min.

### ELISA assay

ELISA kits for GZMB (RK00089, Abclonal), IFNγ (RK00015, Abclonal), TNFα (RK00030, Abclonal) were used for detection. Supernatant samples were stored in an ultra-low temperature freezer, and all procedures were carried out according to the manufacturer’s instructions.

### Immunofluorescence of cells and PDOCO

T cells were dropped onto gelatin-precoated glass slides and allowed to air-dry and adhere naturally. After adhesion, the cells were fixed and permeabilized. The slides were blocked with 3% BSA at room temperature for 1 h, followed by incubation with the primary antibody at 4 °C overnight. The next day, the slides were incubated with the corresponding fluorescent secondary antibody and DAPI (G1012, Servicebio). After incubation, an anti-fade mounting medium was applied, and the slides were sealed for confocal imaging.

For PDOCO, staining was performed using the whole-mounting method. Under a stereomicroscope, organoids of appropriate size were selected. After fixation, permeabilization, and blocking, the organoids were incubated with the primary antibody at 4 °C overnight. The next day, the corresponding fluorescent secondary antibody was added for staining, followed by DAPI staining to label the nuclei.

The primary antibodies include: anti-GOLGA2/GM130 antibody (1:500; 66662-1-Ig, Proteintech), anti-STING antibody (1:300), PAX8 (1:300; 10336-1-AP, Proteintech), CDH1 (1:300; 3195, CST), Ki67 (1:300; sc-23900, SANTA CRUZ), and PanCK (1:300; sc-8018, SANTA CRUZ). The dilution ratios of the fluorescent secondary antibodies used were as follows: anti-mouse Alexa Fluor 488® Conjugate (1:500; 4408, CST) and anti-rabbit Alexa Fluor 647® Conjugate (1:500; 4414, CST).

### Animal work

Five-week-old female C57BL/6 mice were obtained from Shanghai Jiesijie Laboratory Animal Co., LTD. All experimental mice were kept in SPF-grade animal facilities. 100 μL of a 1:1 mixture of DPBS and matrix gel (G4131, Servicebio), containing 1 × 10^7^ ID8 cells, was injected subcutaneously into the axillary region of the mice. On the 7th day after transplanting ID8 cells, drug administration to the mice began. Both Olaparib and NAM were administered via intraperitoneal injection. The dosage of Olaparib was 50 mg/kg, and NAM was 500 mg/kg, with continuous injections for 20 days. Olaparib was diluted with DMSO and 20% SBE-β-CD (A600388, Sangon) for each use. NAM was first dissolved in PBS. All drugs were prepared immediately before use and kept on ice after preparation. Starting from the 7th day after tumor cell transplantation, the body weight of the mice and the size of the subcutaneously transplanted tumors were measured daily. The tumor size was calculated using the formula: Tumor volume (mm^3^) = (length × width^2^) / 2.

### H&E staining and immunohistochemical staining

The fixed tissues were placed in a tissue cassette and processed in a gradient dehydration machine overnight for dehydration and paraffin embedding. The next day, the paraffin-embedded tissues were sectioned. H&E staining of paraffin sections was carried out in the following steps: dewaxing and rehydration, staining, dehydration, and mounting.

Immunohistochemical staining of paraffin sections was performed using the VECTASTAIN Immunohistochemistry Kit (PK-8501, Vector Laboratories) according to the steps of dewaxing and rehydration, antigen retrieval, blocking of endogenous peroxidase, blocking, primary antibody incubation, biotinylated secondary antibody incubation, HRP incubation, color development, counterstaining, dehydration, and mounting, all following the manufacturer’s instructions. The primary antibodies include: anti-CD69 (1:200) and anti-CD3D (1:200).

### Statistical analysis

Values are represented as mean ± SD. GraphPad Prism 7 was used for unpaired Student’s t-test to compare two groups of means and generate p-values. We used standard designation of p-values throughout the figures (ns: not significant; *p < 0.05; **p < 0.01; ***p < 0.001; ****p < 0.0001).

## Results

### NAMPT-mediated NAD^+^ biosynthesis regulates T cell function in OC microenvironment

In previous studies, we developed a TME scoring algorithm based on immune-related genes and defined OC patients with low TME scores as having a TME in a low immune-response state [24]. Further analysis and screening of factors influencing the TME of OC patients identified a link between NAD^+^ metabolism and antitumor immunity (Fig. S1A). In another prior study, we precisely classified OC patients into three immune subtypes, with each subtype predicting distinct antitumor immune responses [25]. We subsequently analyzed the expression of NAD^+^ anabolic-related genes across these immune subtypes and found that, compared to the C1 subtype (immune-activated), multiple genes involved in NAD^+^ metabolism exhibited significantly reduced expression in the C3 subtype (immune-silent) (Fig. S1B). These follow-up analyses based on previous findings suggest that NAD^+^ metabolism may play a role in modulating the antitumor immune response within the TME of OC patients.

The aforementioned findings were derived from whole-transcriptome data of OC tissues. To further investigate the regulatory relationship between NAD^+^ metabolism and various cellular components within the OC microenvironment, with a specific focus on the differential NAD^+^ biosynthesis between malignant OC cells and T cells, we conducted comprehensive analysis of single-cell RNA sequencing dataset GSE217517 (Fig. 1A, Fig. S1C). We found that non-malignant cells consistently exhibited lower expression of NAD^+^ biosynthesis-related genes compared to their malignant counterparts, suggesting an elevated NAD^+^ demand in OC cells (Fig. S1E). In vitro indirect co-culture experiments demonstrated a significant decrease in NAD^+^ levels in T cells over time, while OC cells maintained stable NAD^+^ levels, indicating that cancer cells competitively deplete NAD^+^ availability in microenvironmental T cells (Fig. 1C).

To more precisely explore the intrinsic relationship between NAD^+^ metabolism and the anti-tumor effects of T cells, we performed cell type annotation on non-malignant cell populations. We found that, compared to malignant cells, the expression of NAMPT, the rate-limiting enzyme in the NAD^+^ salvage synthesis pathway, was significantly reduced in T cells (Fig. S1F). We further clustered the specific subtypes of T cells and identified five distinct T cell subtypes (Fig. 1D, E). We observed that within CD8⁺ T cells, the expression of NAMPT showed an even stronger correlation with T cell anti-tumor functions, including cell activity, cytotoxic ability, and chemotactic capacity (Fig. 1F, Fig. S1G). These findings suggest that NAD⁺ anabolic metabolism plays a role in modulating T-cell anti-tumor responses, particularly in relation to the tumor-killing function of CD8^+^ T cells. Moreover, NAMPT-mediated NAD^+^ levels were found to positively regulate T cell anti-tumor function (Fig. 1G, Fig. S1H). In vitro experiments confirmed that NAMPT significantly increased intracellular NAD^+^ levels in T cells, promoting both T cell proliferation and chemokine receptor expression (Fig. 1H–J, Fig. S2A–C). Furthermore, NAMPT-mediated elevation of NAD^+^ levels enhanced the expression of the cytotoxic factors GZMB, as well as multiple cytotoxic cytokines including IFNγ and TNFα (Fig. 1K, L). NAMPT was additionally found to enhance the expression of T cell infiltration- and activation-related proteins CD3D and CD69, which could be downregulated by the NAMPT inhibitor FK866 (Fig. 1M, N, Fig. S2D). These findings collectively demonstrate the crucial role of NAD^+^ biosynthesis in T cell function, with particular emphasis on the NAMPT-mediated salvage pathway as a potent enhancer of T cell-mediated anti-tumor immunity.

### Exogenous NAM supplementation enhances T cell proliferation, chemotaxis, and anti-tumor activity

**Fig. 2:**
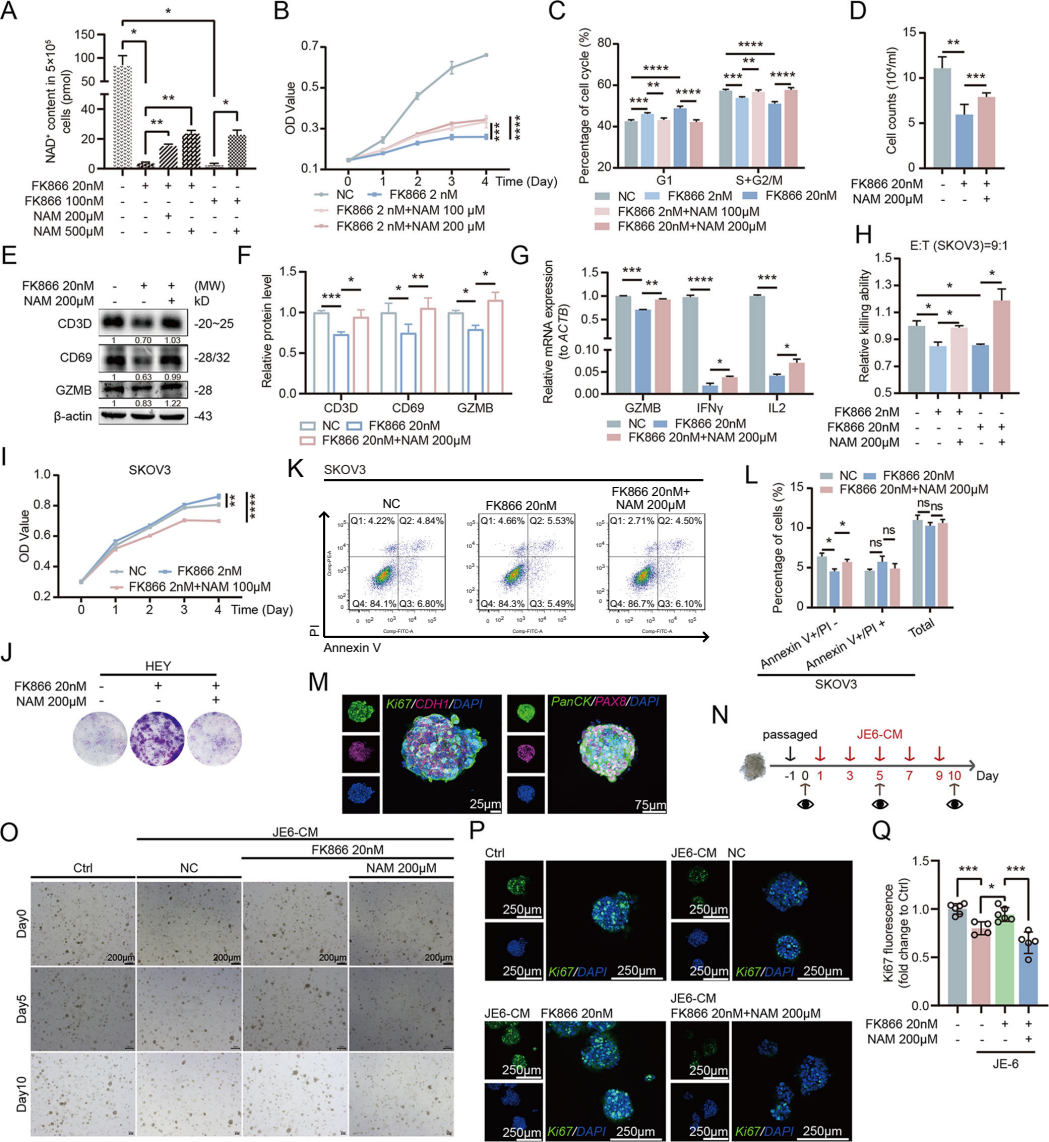
Exogenous NAM supplementation enhances T cell-mediated anti-tumor responses. **A** Exogenous supplementation of NAM in T cells significantly increases NAD^+^ levels that were suppressed by FK866. **B** Decreased NAD^+^ levels inhibit T cell proliferation, while NAM supplementation notably reverses this effect. **C**. NAM increases the number of T cells in the S/G2M phase. **D** Reduced NAD^+^ levels impair T cell chemotaxis, whereas NAM enhances T cell chemotactic ability. **E**, **F** NAM promotes the expression of T cell infiltration and activation markers CD3D, CD69, and GZMB. **G** NAM supplementation upregulates the expression of T cell anti-tumor factors GZMB, IFNγ, and IL2. **H** Reduced NAD^+^ levels in T cells inhibit their cytotoxic ability, while NAM enhances T cell-mediated killing. **I** NAM enhances T cell anti-tumor function, inhibiting the proliferation of SKOV3 cells. **J** NAM enhances T cell anti-tumor function, inhibiting the proliferation of HEY cells. **K**, **L** NAM enhances T cell-mediated killing of OC cells and promotes apoptosis of SKOV3. **M** Construction of PDOCOs and their validation by immunofluorescence staining for Ki67, CDH1, PanCK, and PAX8. **N** Treatment of PDOCOs with T cell supernatant. **O** Increased NAD^+^ levels in T cells enhance their inhibitory effect on PDOCOs growth. **P**, **Q** NAM enhances the inhibitory effect of T cells on PDOCOs growth, with a significant decrease in Ki67 expression.

Compared to genetic manipulation of T cells, exogenous supplementation with small molecules that boost NAD^+^ levels offers greater convenience. In mammalian cells, the salvage pathway serves as the primary route for NAD^+^ biosynthesis, utilizing three NAD^+^ precursors: NAM (nicotinamide), NMN (nicotinamide mononucleotide), and NR (nicotinamide riboside). We initially evaluated the effects of these three NAD^+^ precursors on OC cells to ensure that exogenous NAD^+^ supplementation would not promote tumor progression. Our results indicated that neither NAM nor NR significantly stimulated OC cell growth (Fig. S2E). Previous studies have reported that NAM exhibits superior cellular uptake efficiency compared to NR [26], leading us to prioritize NAM as the NAD^+^-boosting small molecule for our investigation. Our study demonstrating that NAM effectively elevates NAD^+^ levels and attenuates FK866-induced NAD^+^ depletion (Fig. 2A). Further experiments revealed that NAM supplementation reverses FK866-mediated suppression of T cell proliferation (Fig. 2B, Fig. S2F, G). NAM treatment alleviated G1 phase arrest in T cells, leading to increased cell populations in S and G2/M phases, suggesting that NAM-induced NAD^+^ elevation promotes T cell proliferation through mechanisms analogous to NAMPT-mediated effects (Fig. 2C, Fig. S2H, I). Moreover, NAM-mediated NAD^+^ restoration significantly reversed FK866-impaired chemotactic capacity and re-established the expression of chemokine receptors CCR4, CCR5, and CCR9 (Fig. 2D, Fig. S2J).

The FK866-mediated NAD^+^ depletion significantly reduced the expression levels of CD3D, CD69, and GZMB in T cells, while NAM supplementation effectively reversed this suppression of T cell activation (Fig. 2E, F, Fig. S3A, B). The expression of T cell activation receptor IL2R, along with various anti-tumor secretory factors (GZMB, IFNγ) and the inflammatory cytokine IL2, showed strong correlation with intracellular NAD^+^ levels (Fig. 2G, Fig. S3C, D). To further investigate the impact of elevated NAD⁺ levels in T cells on their tumor-killing efficacy, we conducted luciferase-based cytotoxicity assays. The results demonstrated that inhibition of NAD^+^ biosynthesis in T cells attenuated their tumoricidal activity, whereas NAM supplementation significantly enhances the cytotoxic capacity of T cells against OC cells (Fig. 2H, Fig. S3E–G). Furthermore, NAM-mediated NAD^+^ elevation substantially augmented the secretion of anti-tumor factors from T cells, leading to significant inhibition of proliferation and increased apoptosis in co-cultured OC cells (Fig. 2I–L, Fig. S3H–J). Patient-derived organoid, as an emerging model system, retain genetic lineages and pathological characteristics similar to their parental tumors, thereby accurately recapitulating the complexity, microarchitecture, and growth patterns of tumors in vivo. Here, we established PDOCO models (Fig. 2M). Consistent with the cell line models, we co-cultured PDOCOs with NAM-treated T cells and observed significantly reduced Ki67 intensity, demonstrating that NAM enhances the inhibitory effect of T cells on tumor proliferation (Fig. 2N–Q). Moreover, T cell exhaustion represents a critical factor contributing to poor anti-tumor functionality despite T cell infiltration, and alleviating this exhausted state is key to reversing “cold” immune microenvironments. Our findings also demonstrate that NAM can ameliorate NAD^+^ deficiency-induced T cell exhaustion phenotypes (Fig. S3K–M). These results collectively underscore the crucial role of NAD^+^ levels in maintaining T cell anti-tumor functionality and establish NAM as an effective NAD^+^ precursor for enhancing T cell-mediated anti-tumor responses.

### The p-STING/p-IRF3 axis plays a crucial role in NAD^+^-mediated activation of T cell function and anti-tumor immunity

**Fig. 3:**
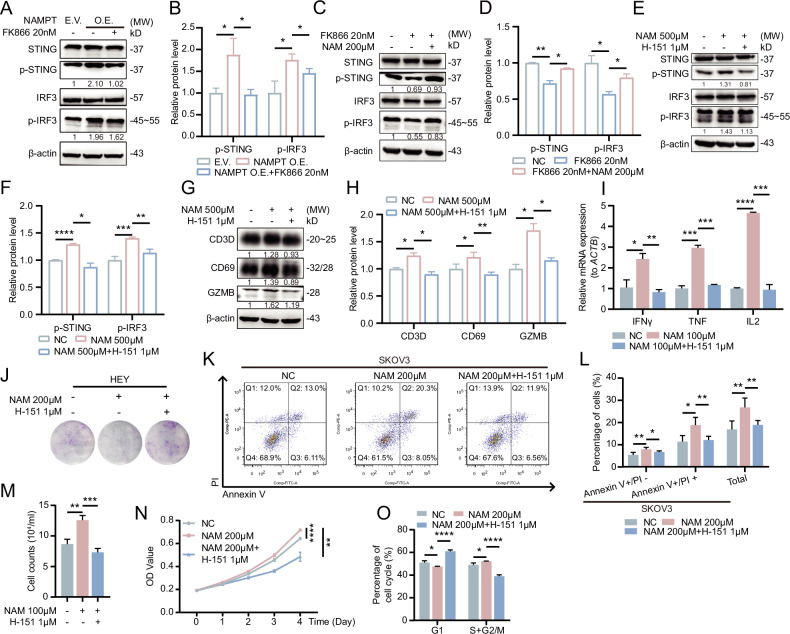
The p-STING/p-IRF3 axis plays a crucial role in NAD^+^-mediated activation of T cell function and anti-tumor immunity. **A**, **B** NAMPT promotes the activation of the p-STING/p-IRF3 axis in T cells. **C-D**. Reduced NAD^+^ levels inhibit the activation of the p-STING axis, while NAM promotes the activation of the p-STING/p-IRF3 axis in T cells. **E**, **F** Inhibition of STING significantly attenuates the NAM-promoted activation of downstream p-IRF3. **G**, **H** Inhibition of STING in T cells markedly reduces the expression of NAM-activated anti-tumor effector proteins. **I** Inhibition of STING in T cells significantly downregulates the expression of NAM-activated anti-tumor factors. **J–L** Inhibition of STING in T cells significantly weakens the NAM-enhanced cytotoxic ability of T cells. **J** After STING inhibition in T cells, the NAM-promoted inhibitory effect of T cells on the proliferation of HEY is significantly reduced. **K**, **L** After STING inhibition in T cells, the number of apoptotic SKOV3 decreases. **M** STING inhibition diminishes the NAM-promoted enhancement of T cell chemotaxis. **N**, **O** STING inhibition weakens the NAM-promoted proliferation of T cells, reducing the number of cells in the S/G2M phase.

Targeting the STING signaling pathway has emerged as a promising strategy for enhancing anti-tumor immunity. While recent studies have gradually elucidated the functional significance of STING signaling in T cells, the role of endogenous STING pathway activation in T cell responses remains largely unexplored. In this study, we demonstrate that both NAMPT-mediated and NAM-induced elevation of intracellular NAD^+^ levels effectively activate the p-STING/p-IRF3 signaling axis (Fig. 3A–D, Fig. S4A, B).

To clarify that the NAD^+^ level-mediated activation of the p-STING axis directly acts on STING, we further investigated the inhibitory effects of the cGAS inhibitor RU.521 and the STING inhibitor H-151 on NAM-mediated p-STING axis activation. The results showed that H-151 significantly inhibited NAM-activated p-STING, while RU.521 had a weaker inhibitory effect on the STING axis activation induced by NAM. (Fig. 3E, F, Fig. S4C). Furthermore, H-151 not only inhibited the p-STING/p-IRF3 axis but also significantly reduced the expression of T cell infiltration and activation markers (CD3D and CD69) as well as anti-tumor factors (GZMB, IFNγ, and TNFα) (Fig. 3G–I, Fig. S4D).

Moreover, inhibition of the STING axis significantly suppressed the NAM-mediated enhancement of T cell cytotoxic activity, as evidenced by a reduction in the inhibitory effect on OC cell proliferation and a decrease in OC cell apoptosis (Fig. 3J–L, Fig. S4E). H-151 not only diminished NAMPT-promoted T cell proliferation (Fig. S2A) but also inhibited NAM-induced enhancement of T cell proliferation and chemotactic capacity (Fig. 3M–O, Fig. S4F–J). These findings collectively indicate that NAD^+^-mediated augmentation of T cell proliferation, chemotaxis, and anti-tumor cytotoxicity is dependent on the activation of the endogenous STING signaling axis.

### Elevated NAD^+^ levels downregulate SURF4 to promote p-STING axis activation

**Fig. 4:**
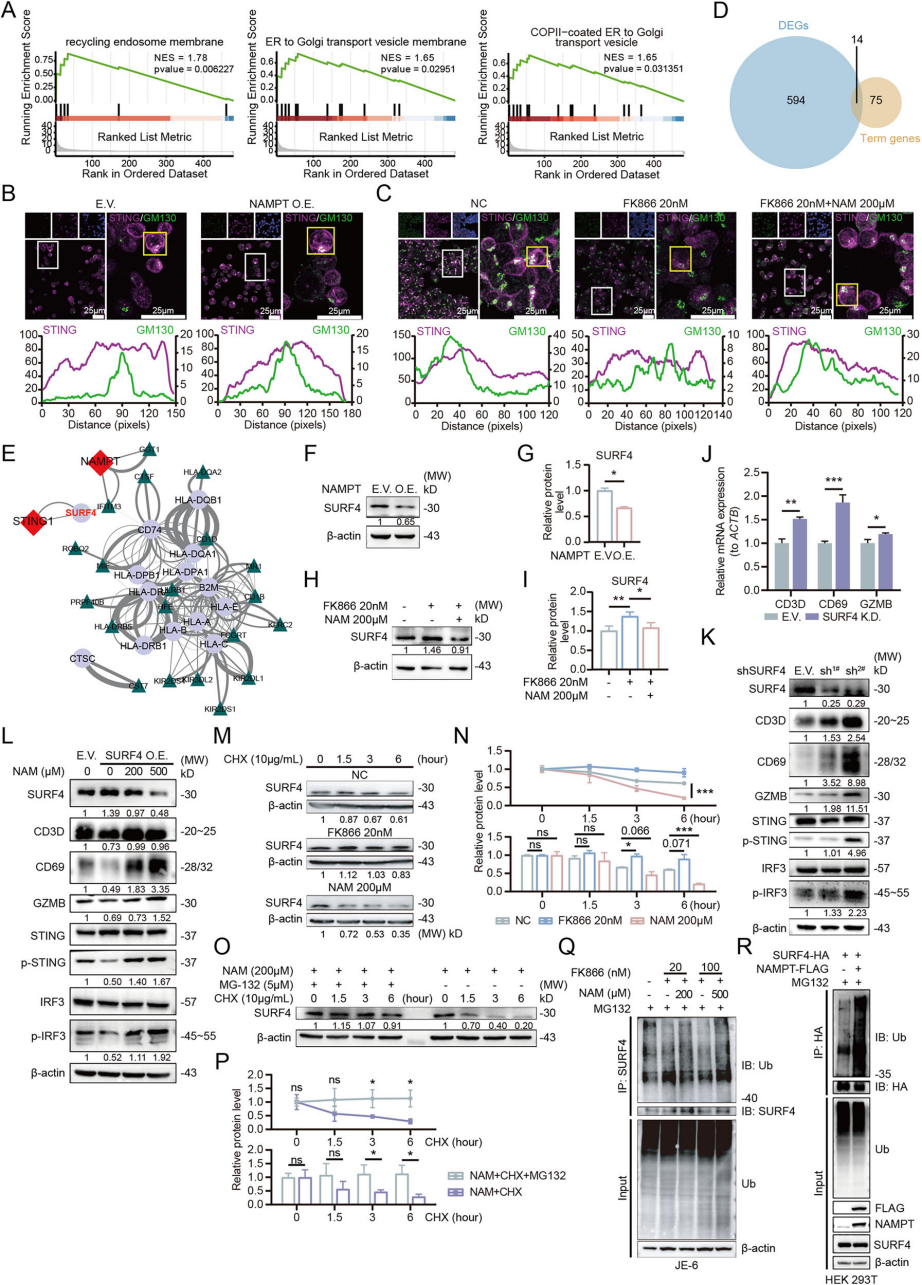
Elevated NAD^+^ levels promote ubiquitination-mediated degradation of SURF4. **A** The NAD^+^ level in T cells regulates protein transport between the ER and the Golgi apparatus. **B** Overexpression of NAMPT promotes the aggregation of STING on the Golgi apparatus in T cells. **C** Reduced NAD^+^ levels decrease the aggregation of STING on the Golgi apparatus in T cells, while NAM promotes the re-aggregation of STING on the Golgi apparatus. **D** Identification of key molecules related to ER-Golgi transport and NAD^+^ metabolism. **E** Identifying SURF4 as a key protein via PPI analysis. **F**, **G** Overexpression of NAMPT downregulates SURF4 in T cells. **H**, **I** Reduced NAD^+^ levels increase SURF4 expression, while NAM supplementation downregulates SURF4. **J** Downregulation of SURF4 promotes the expression of infiltration and activation marker genes. **K** Knockdown of SURF4 activates the p-STING/p-IRF3 axis in T cells and upregulates the expression of anti-tumor proteins. **L**. Overexpression of SURF4 inhibits the activation of the p-STING/p-IRF3 axis and downregulates the expression of infiltration and activation proteins, while NAM reverses these effects by downregulating SURF4. **M**, **N** The degradation rate of SURF4 protein slows down when NAD^+^ levels are reduced, while NAM accelerates SURF4 protein degradation. **O**, **P** NAM promotes the ubiquitination-dependent degradation of SURF4 protein. **Q** NAM supplementation increases the abundance of ubiquitinated SURF4 protein in T cells. **R** Overexpression of NAMPT, mediated by changes in NAD^+^ levels, increases the abundance of ubiquitinated SURF4 protein in HEK293T cells.

Following cellular stimulation by external factors such as tumor-derived DNA, intracellular cGAS protein becomes activated, triggering the translocation of STING protein from the endoplasmic reticulum (ER) to the Golgi apparatus, where it undergoes phosphorylation and subsequently activates downstream p-TBK and p-IRF3, ultimately leading to interferon signaling transcription [27]. Throughout this process, STING undergoes ER-Golgi trafficking and completes its functional activation at the Golgi apparatus [28, 29]. We discovered that NAMPT-mediated NAD^+^ levels significantly influence ER-Golgi transport, particularly protein trafficking from the ER to the Golgi (Fig. 4A). Consequently, we examined the intracellular localization of STING protein in T cells and found that NAMPT overexpression resulted in increased STING accumulation at the Golgi apparatus compared to controls (Fig. 4B). Conversely, FK866-mediated NAD^+^ depletion reduced STING localization at the Golgi, while NAM supplementation restored STING accumulation at the Golgi (Fig. 4C). These findings suggest that intracellular NAD^+^ levels influence STING retention at the Golgi apparatus, indicating that NAD^+^ level alterations may affect STING intracellular trafficking.

Intracellular protein transport relies on vesicles, and due to the unique structure of STING, it cannot directly bind to vesicles and requires intermediary mediators [30]. To identify proteins influencing STING Golgi retention, we screened NAMPT-associated differentially expressed genes and GSEA-enriched ER-Golgi pathway genes, ultimately identifying 14 potential candidates (Fig. 4D). Protein-protein interaction prediction analysis revealed that SURF4 not only potentially interacts with STING but is also indirectly regulated by NAMPT, suggesting its potential role in NAD^+^-mediated p-STING axis activation (Fig. 4E). In NAMPT-overexpressing T cells, SURF4 protein levels were downregulated (Fig. 4F, G). Conversely, FK866-mediated NAD^+^ depletion increased intracellular SURF4 expression, which was reversed by NAM supplementation (Fig. 4H, I). These results demonstrate that SURF4 protein expression is significantly influenced by NAD^+^ levels.

Recent studies have reported that SURF4 recruits STING, facilitating its retrograde transport from the Golgi to the ER, while SURF4 knockdown results in STING accumulation at the Golgi [31]. In certain contexts, SURF4-mediated signal termination may prevent excessive immune activation [32]. Protein docking analysis revealed that the docking model between STING and SURF4 suggests a highly efficient binding interaction (Fig. S5A, Table S4). Furthermore, we detected a specific binding between STING and SURF4 proteins in both JE-6 T cells and HEK 293 T cells (Fig. S5B, C). SURF4 knockdown in T cells resulted in elevated CD3D, CD69, and GZMB expression, along with significant activation of the p-STING/p-IRF3 axis (Fig. 4J, K, Fig. S5D). Conversely, SURF4 overexpression in T cells significantly reduced the expression of activation markers (CD3D, CD69) and anti-tumor proteins (GZMB), while simultaneously suppressing the p-STING/p-IRF3 axis (Fig. 4L). However, NAM supplementation reversed SURF4-mediated suppression of activation markers and reactivated the p-STING/p-IRF3 axis (Fig. 4L). These collective findings demonstrate that SURF4 participates in NAD^+^-mediated regulation of the p-STING/p-IRF3 axis and downstream T cell activation. Elevated NAD^+^ levels suppress SURF4 expression, promoting STING stabilization at the Golgi apparatus and thereby enhancing p-STING/p-IRF3 axis-mediated T cell activation.

### Elevated NAD^+^ levels promote ubiquitination-mediated degradation of SURF4

To investigate the regulatory mechanism of NAD^+^ on SURF4 expression, we examined SURF4 transcriptional levels under different NAD^+^ conditions. Our results demonstrated that NAD^+^ levels did not affect SURF4 transcription (Fig. S5E, F). Through protein synthesis inhibition experiments, we observed that reduced NAD^+^ levels significantly slowed SURF4 protein degradation in T cells, while NAM-mediated NAD^+^ elevation accelerated SURF4 degradation, suggesting that NAD^+^ levels regulate SURF4 expression through post-translational modification of protein stability (Fig. 4M, N).

The protein modification database suggests that SURF4 protein can undergo various post-translational modifications, with ubiquitination being one that has been predicted and reported in multiple studies (Fig. S5G). We therefore focused on NAD^+^-mediated ubiquitination of SURF4. Proteasome inhibitor MG-132 treatment significantly attenuated NAM-induced SURF4 degradation, supporting the involvement of ubiquitin-proteasome system (Fig. 4O, P). Further examination of SURF4 ubiquitination under different NAD^+^ conditions revealed that NAD^+^ depletion significantly reduced SURF4 ubiquitination in T cells, while NAM supplementation increased ubiquitination levels (Fig. 4Q). Similarly, in HEK 293 T cells, NAMPT overexpression enhanced SURF4 ubiquitination (Fig. 4R). To further investigate the regulatory factors involved in NAM-mediated SURF4 ubiquitination in T cells, we analyzed the interactions among NAMPT, SURF4, E3 ubiquitin ligases, and deubiquitinating enzymes (DUBs). Our analysis revealed correlations between the expression of NAMPT/SURF4 and both the E3 ubiquitin ligase PELI1 and the deubiquitinase USP47 (Fig. S5H, I). External dataset GSE53772 demonstrated that FK866 treatment upregulates USP47 expression, while in vitro experiments confirmed that NAM supplementation suppresses USP47 expression (Fig. S5J, K). Single-cell RNA sequencing analysis of OC showed an inverse correlation between USP47 expression and various antitumor factors within T cell populations (Fig. S5L).

These findings collectively demonstrate that both NAMPT- and NAM-mediated NAD^+^ elevation promotes ubiquitination-mediated degradation of SURF4 protein, potentially through regulation of USP47. This downregulation of SURF4 enables STING protein retention at the Golgi apparatus, subsequently activating the downstream p-IRF3 axis and interferon signaling, ultimately enhancing T cell-mediated anti-tumor immunity.

### Enhancing T cell NAD^+^ levels potentiates anti-tumor immunity during PARPi treatment in OC

The activation of STING in immune cells primarily occurs through the acquisition of exogenous tumor DNA or tumor-derived cGAMP [33, 34]. PARP inhibitors (PARPi) induce DNA damage by preventing the repair of single-strand breaks, resulting in the accumulation of double-stranded DNA (dsDNA) that not only activates the cGAS-cGAMP pathway but is also released into the TME upon cell death [35]. The immunomodulatory effects mediated by PARPi-induced dsDNA generation contribute to their therapeutic efficacy independent of BRCA mutation status, with their effectiveness requiring intact T cell-mediated immune responses [36]. Therefore, we investigated the potential of enhancing T cell NAD^+^ levels to augment anti-tumor immunity during PARPi treatment at both cellular and animal levels.

**Fig. 5:**
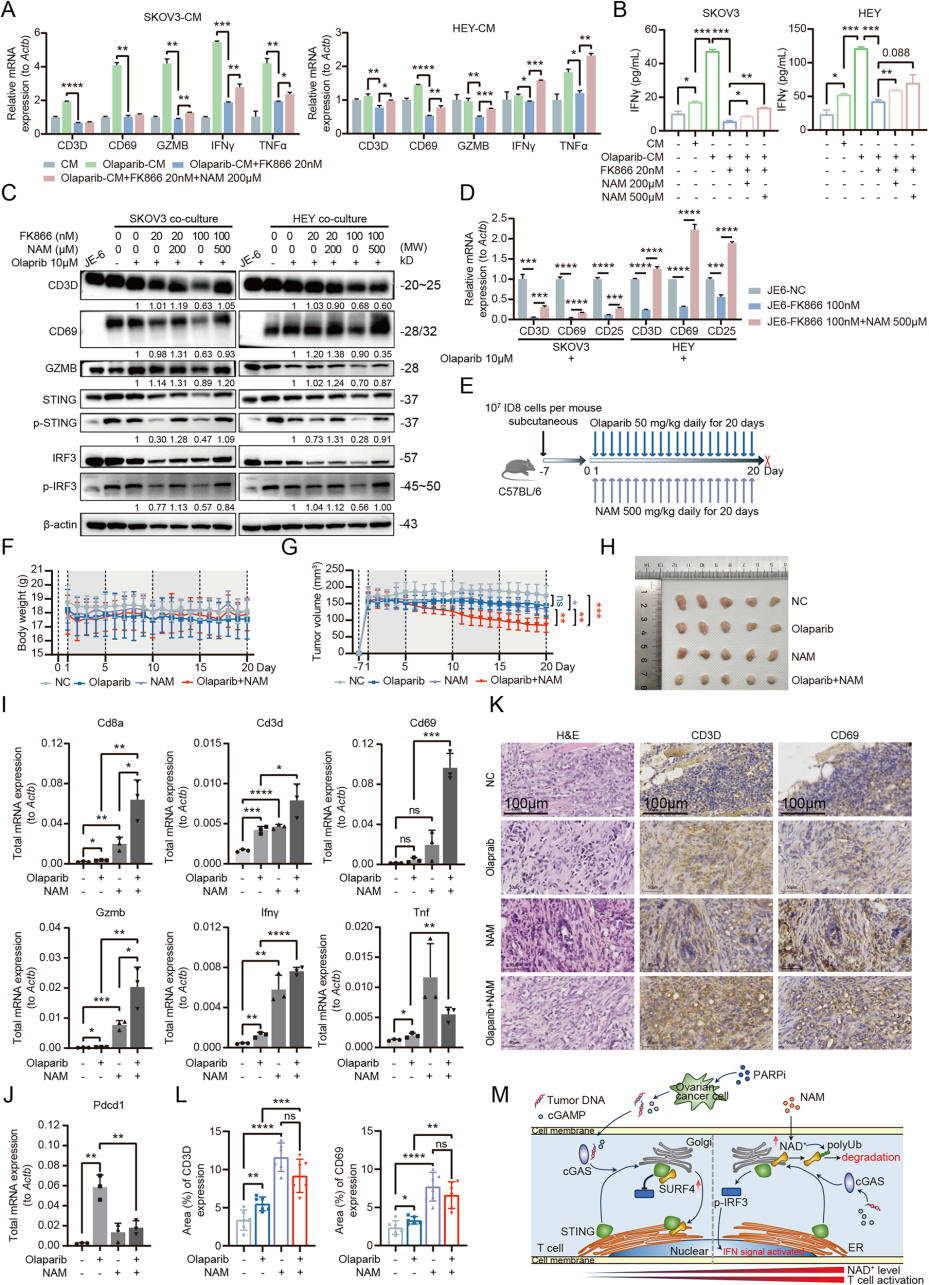
Enhancing T cell NAD^+^ levels potentiates anti-tumor immunity during PARPi treatment in OC. **A** The supernatant of OC cells treated with Olaparib can activate the expression of anti-tumor-related proteins in T cells. Low NAD^+^ levels in T cells weaken the Olaparib-OC cell-mediated activation of T cells, which can be reversed by NAM supplementation. **B** Olaparib-treated OC cells activate IFNγ secretion in T cells, and the secretion level is regulated by the basal NAD^+^ content in T cells. Sufficient NAD^+^ levels promote IFNγ secretion in stimulated T cells. **C**, **D** Direct co-culture of OC cells and T cells further confirms that the anti-tumor activity of T cells mediated by Olaparib-treated OC cells is influenced by NAD^+^ levels. Low NAD^+^ levels inhibit T cell activation, while NAM supplementation enhances the anti-tumor function of T cells. **E** Schematic diagram of the combined treatment regimen of Olaparib and NAM in a subcutaneous tumor mouse model. **F** No significant changes in body weight were observed in any treatment group during the treatment period. **G**, **H** The subcutaneous tumors in the Olaparib-NAM combination treatment group showed significant shrinkage, with effects significantly superior to those of the Olaparib monotherapy or NAM monotherapy groups (*n* = 5 per group). **I** The expression levels of T cell anti-tumor-related genes (Cd8a, Cd3d, Cd69, Gzmb, Ifnγ, and Tnf) were significantly upregulated in the subcutaneous tumors of the Olaparib-NAM combination treatment group. **J** The expression of the T cell exhaustion marker gene Pdcd1 was significantly reduced in the subcutaneous tumors of the Olaparib-NAM combination treatment group. **K**, **L** Immunohistochemistry showed significantly increased expression of CD3D and CD69 in the subcutaneous tumors of the Olaparib-NAM combination treatment group. **M** Schematic diagram summarizing the research model of this study.

Our findings revealed that Olaparib alone did not directly activate the p-STING/p-IRF3 axis or downstream anti-tumor activity in T cells (Fig. S6A). However, supernatant from Olaparib-treated OC cells significantly activated the p-STING/p-IRF3 axis in T cells, accompanied by upregulation of infiltration marker CD3D, activation marker CD69, and cytotoxic factor GZMB (Fig. 5A, B, Fig. S6B, D). These results indicate that Olaparib requires OC cell mediation to activate T cell anti-tumor function in the TME. Furthermore, the enhancement of T cell anti-tumor activity mediated by Olaparib-treated OC cells was dependent on intracellular NAD^+^ levels, as NAD^+^ depletion in T cells significantly inhibited this activation (Fig. 5A, B, Fig. S6C, D). Direct co-culture experiments of T cells with OC cells demonstrated that Olaparib-treated cancer cells only promoted T cell anti-tumor function when T cells maintained high NAD^+^ levels (Fig. 5C, D). Intracellular localization analysis revealed that STING localization in T cells was highly sensitive to NAD^+^ level fluctuations when mediated by Olaparib-treated OC cells, emphasizing the importance of Golgi-localized STING for downstream p-STING/p-IRF3 axis activation and enhanced anti-tumor function following exogenous stimulation (Fig. S6E).

We found pre-treatment of T cells with NAM, combined with Olaparib, significantly inhibited PDOCO growth and induced cell death, as evidenced by markedly reduced Ki67 expression, demonstrating the efficacy of NAM in potentiating Olaparib-induced T cell activation (Fig. S6F, G). In vivo studies showed that while neither Olaparib nor NAM alone significantly reduced subcutaneous tumor growth in mice, their combination resulted in substantial tumor regression, suggesting NAM as a potential adjuvant to enhance PARPi efficacy (Fig. 5E–H). Tumors from the combination treatment group exhibited significantly increased expression of Cd8a, Cd3d, Cd69, Gzmb, Ifnγ, and Tnf, along with downregulation of the immune checkpoint gene Pdcd1 (Fig. 5I–L). These results highlight the potential of NAM as an adjuvant to enhance T cell-mediated anti-tumor immunity during PARPi treatment in OC.

## Discussion

The complexity of the OC microenvironment significantly impacts patient outcomes. A comprehensive analysis of this microenvironment facilitates understanding of inter-component interactions and identification of regulatory factors affecting anti-tumor immunity, thereby enabling development of novel therapeutic strategies [37, 38]. Our previous research characterized the anti-tumor immune status in OC microenvironment, revealing that most patients exhibit an immune-silent microenvironment [25]. This immunosuppressive state not only limits anti-tumor immune cell function but also reduces immunotherapy efficacy, accelerates chemotherapy resistance, and promotes tumor progression [9].

Our earlier findings suggested NAD^+^ metabolism might regulate the immune-silent state in OC microenvironment [24]. As a fundamental biological element, NAD^+^ plays crucial roles in energy metabolism, oxidative stress, and aging [39]. Previous studies have demonstrated that tumor cells exhibit higher NAD^+^ demands compared to non-tumor cells, a phenomenon dependent on the Warburg effect, where tumor cells must increase NAD^+^ levels to support high glycolytic activity [40]. However, this tumor-specific metabolic alteration may compromise NAD^+^ availability for other immune components in the microenvironment [41, 42]. Through co-culture experiments, we confirmed that OC cells restrict NAD^+^ availability for T cells, while T cell NAD^+^ levels are closely associated with their anti-tumor functionality. T cell activation depends on NAD^+^ for energy production and signal transduction, and NAD^+^ depletion may lead to T cell exhaustion, characterized by reduced proliferation, cytokine production, and cytotoxic activity. Previous research has shown that NAD^+^ supplementation significantly enhances CAR-T cell anti-tumor activity in vitro and prolongs survival in animal models of CAR-T therapy and immune checkpoint blockade, suggesting NAD^+^ may improve T cell-based immunotherapy efficacy [43–45]. In this study, we found that elevating T cell NAD^+^ levels promotes proliferation, upregulates chemokine receptor expression, and enhances chemotactic capacity. Furthermore, increased NAD^+^ levels promote expression of T cell infiltration and activation markers, stimulate anti-tumor factor secretion, and enhance tumor cell killing. NAM, as an NAD^+^ precursor, effectively enhances T cell anti-tumor function. Although NAM conversion to NAD^+^ depends on NAMPT activity, studies have shown that NAM can reverse FK866-induced cytotoxicity [46]. Our research demonstrated that NAM restores FK866-suppressed NAD^+^ levels and reverses FK866-inhibited T cell anti-tumor activity. Importantly, exogenous NAM supplementation does not promote OC progression [47, 48]. These findings suggest that targeting NAD^+^ metabolism represents a promising therapeutic strategy, potentially reprogramming the TME metabolic landscape and restoring immune cell function through promoting NAD^+^ biosynthesis, reducing NAD^+^ consumption, or supplementing NAD^+^ precursors, ultimately improving OC patient outcomes.

The cGAS-STING axis serves as a crucial regulator of cancer immunity, with its mediated immune-supportive microenvironment potentially inhibiting malignant progression [49]. Although STING is generally believed to function by bridging innate and adaptive immunity upon activation, increasing evidence suggests that adaptive immune cells can directly respond to STING engagement, with STING activation in adaptive immune cells producing higher IFN levels than in innate immune cells [50, 51]. Previous studies have indicated that intracellular STING signaling promotes stem-like CD8^+^ T cell maintenance, suggesting T cell STING activation enhances anti-tumor immune responses [52]. We found that elevating T cell NAD^+^ levels activates the STING axis and downstream p-IRF3 signaling, thereby enhancing T cell anti-tumor function. Previous research on NAD^+^ regulation of STING signaling has primarily focused on cellular senescence. Contrary to our findings, studies by Hou et al. and Myakala K et al. demonstrated that reduced NAD^+^ levels mediate STING axis activation, inducing pro-inflammatory factor expression and cellular senescence, while NR supplementation alleviates this pro-inflammatory microenvironment and slows Alzheimer’s disease progression [53, 54]. However, other studies have shown that NAD^+^ can induce pro-inflammatory microenvironments, promoting macrophage M2-to-M1 polarization or tumor growth [15, 47, 55]. These discrepancies may result from differences in disease models.

STING axis-mediated interferon signaling activation depends on STING protein conformational changes following cGAMP binding, subsequent STING translocation from ER to Golgi, and downstream IRF3 signaling activation at the Golgi, leading to IRF3 phosphorylation and nuclear translocation for interferon pathway activation [56]. We found that NAD^+^ levels regulate intracellular protein transport, particularly ER-Golgi trafficking. Deng et al. reported that impaired STING transport between ER and Golgi leads to sustained autoimmune activation, suggesting Golgi localization is crucial for STING downstream signaling activation [32]. Through co-localization experiments, we demonstrated that increased NAD^+^ levels promote STING stabilization at the Golgi, potentially representing a key mechanism for NAD^+^-enhanced T cell anti-tumor immunity. By screening NAD^+^-regulated genes involved in ER-Golgi protein transport, we identified SURF4 as a key player in this process. Previous studies have demonstrated SURF4’s robust protein transport capabilities, particularly in mediating Golgi-to-ER retrograde transport. Mukai K et al. first proposed in 2021 that SURF4 mediates STING retrograde transport to ER, affecting STING Golgi localization [31]. We confirmed SURF4-STING interaction in T cells and demonstrated that SURF4 downregulation activates T cell STING axis and downstream anti-tumor immunity. Furthermore, T cell SURF4 expression is regulated by NAD^+^ levels, with increased NAD^+^ downregulating SURF4. Thus, NAD^+^ regulation of STING axis activation in T cells is mediated by SURF4. Previous studies have shown that NAD^+^ levels can affect post-translational modifications such as acetylation and PARylation by regulating catabolic enzymes PARPs and SIRTs [57, 58]. Mass spectrometry studies have predicted SURF4 ubiquitination [59]. In this study, we confirmed NAD^+^-mediated SURF4 ubiquitination through IP experiments. Collectively, increased T cell NAD^+^ levels promote SURF4 ubiquitination and degradation, reducing STING binding and retrograde transport, thereby stabilizing STING at the Golgi and enhancing T cell interferon signaling activation.

Immune cell STING activation depends on acquisition of exogenous tumor DNA or tumor-derived cGAMP [34]. Recent studies have shown that PARPi-induced dsDNA generation mediates immune response regulation, contributing to BRCA mutation-independent therapeutic effects, with efficacy requiring intact T cell-mediated immune responses [36]. We found that PARPi Olaparib does not directly activate T cells but can activate T cells through OC cell mediation, regulated by T cell NAD^+^ levels. NAM-Olaparib combination therapy significantly enhanced OC growth inhibition and increased intratumoral T cell activation in mouse models. These results suggest that NAM as an adjuvant can enhance Olaparib efficacy by increasing T cell NAD^+^ levels, further activating the STING axis, thereby enhancing T cell anti-tumor function, providing a novel strategy for OC treatment.

However, the clinical application of NAM still presents several challenges. A study demonstrated that after a single 200 mg oral dose of NAM, the plasma concentration peaked at 0.5 h and subsequently declined, while NAD^+^ levels reached maximum concentration at 12 hours [60]. Furthermore, high-dose NAM (3–6 g) administration significantly elevated NAD^+^ levels but also induced adverse effects including nausea and vomiting [61]. These pharmacokinetic characteristics indicate that NAM dosing regimens require careful optimization to maintain effective drug concentrations while minimizing side effects. Although NAM-PARPi combination enhances anti-tumor efficacy, their overlapping toxicity profiles may contribute to hematologic adverse events. The combination could exacerbate leukopenia and thrombocytopenia, potentially increasing risks of infection and bleeding [62, 63]. Additionally, while NAM activates the STING pathway to enhance immune responses, its combination with PARPi may disrupt immune cell balance in the tumor microenvironment by promoting regulatory T cell (Treg) proliferation, thereby potentially compromising anti-tumor immunity [64]. Consequently, future studies should focus on optimizing NAM dosing regimens and investigating individual variations in response to NAM-PARPi combinations to improve both efficacy and safety in ovarian cancer treatment.

This study has several limitations that need to be addressed. In the mechanistic investigation, the regulation of SURF4 ubiquitination mediated by NAM-induced NAD^+^ elevation requires further clarification in subsequent studies. Through bioinformatics analysis, we identified the deubiquitinase USP47 as being associated with NAD^+^ levels and correlated with various anti-tumor genes in ovarian cancer microenvironment T cells. Therefore, future research will focus on elucidating the detailed mechanisms by which NAD^+^ levels regulate SURF4 ubiquitination. Additionally, while protein docking has predicted multiple potential interaction sites between SURF4 and STING, the specific binding sites between these two proteins need to be experimentally validated in follow-up studies. Finally, the preclinical animal experiments combining NAM with PARPi require further refinement, and the immunostimulatory effects of NAM need to be evaluated through additional experiments.

In conclusion, this study reveals the crucial role of NAD^+^ metabolism in regulating T cell function, with NAD^+^ increase promoting SURF4 ubiquitination and degradation, thereby stabilizing STING at the Golgi and enhancing T cell anti-tumor effects. Furthermore, this study provides theoretical basis for NAM as an adjuvant to enhance PARPi efficacy (Fig. 5M). These findings offer new perspectives and potential therapeutic strategies for OC immunotherapy, potentially improving patient outcomes and treatment efficacy.

## Supplementary information

Supplementary Information

Supplementary Figure 1

Supplementary Figure 2

Supplementary Figure 3

Supplementary Figure 4

Supplementary Figure 5

Supplementary Figure 6

Supplementary Table 1

Supplementary Table 2

Supplementary Table 3

Supplementary Table 4

Original Western Blot Images

## Data Availability

All data and computer code analyzed in this research are available from the corresponding author on reasonable request. Original western blots images are provided in Supplementary Materials.
