# Supplementary material for: NAD+ Metabolism-Mediated SURF4-STING Axis Enhances T-Cell Anti-Tumor Effects in the Ovarian Cancer Microenvironment
Source: Cell Death Dis. 2025 Aug 23;16(1):640. doi: 10.1038/s41419-025-07939-9 (PMC12373823; doi:10.1038/s41419-025-07939-9)
Supplement: Supplementary file 1 — Supplementary Information [file 41419_2025_7939_MOESM1_ESM.docx]

**Supplementary information**

**NAD^+^ Metabolism-Mediated SURF4-STING Axis Enhances T-Cell Anti-Tumor Effects in the Ovarian Cancer Microenvironment**

Jiacheng Shen^1^, Fangfang Xu^1^, Tingwei Liu^1^, Yingjun Ye^1^, Shaohua Xu^1,*^

^1^Department of Gynecology, Shanghai Key Laboratory of Maternal Fetal Medicine, Shanghai Institute of Maternal-Fetal Medicine and Gynecologic Oncology, Shanghai First Maternity and Infant Hospital, School of Medicine, Tongji University, Shanghai, China

^*^Correspondence author: Shaohua Xu (xushaohua@tongji.edu.cn)

**Supplementary Methods**

**Source information of single-cell sequencing data for OC**

The single-cell sequencing data of OC used in this study were obtained from the research articles by the Greene CS research group [1, 2]. According to their study, the samples for single-cell sequencing were collected from 8 patients with high-grade serous ovarian cancer (HGSOC) by the Ovarian Cancer Research Center Tumor Bio-specimen Bank at the University of Pennsylvania. All patients underwent primary debulking surgery and did not receive neoadjuvant chemotherapy.

**Establishment and Culture of PDOCOs**

1) Establishment and Culture of PDOCOs

OC tissues obtained from clinical sources should be processed within 24 hours to ensure organoid viability. For short-term storage, tissues can be preserved in Advanced DMEM/F12 (AdDF12) supplemented with 5 μM Y-27632. The detailed protocol for PDOCO isolation is as follows:

i. Tissue Dissociation: Wash OC tissues several times with DPBS until no visible blood remains. Mince the tissues into 1 mm^3^ fragments using sterile scissors. Digest the fragments in AdDF12 containing 10 U/mL DNase, 5 μM Y-27632, and 500 U/mL Collagenase I at 150 rpm in a 37℃ incubator for 20 min.

ii. Neutralization and Filtration: After digestion (until no visible tissue fragments remain), neutralize the mixture with an equal volume of FBS. Filter the suspension through a 100 μm cell strainer.

iii. Red Blood Cell Lysis: Centrifuge at 300 ×g and discard the supernatant. Resuspend the pellet in red blood cell lysis buffer, gently invert at room temperature, and centrifuge at 300 ×g for 3 min. Repeat if necessary until the pellet appears free of red coloration.

iv. Embedding in BME Matrix: Wash once with AdDF12 and remove residual medium. Keep the tube on ice, resuspend the cell pellet in an appropriate volume of BME matrix, and plate 20 µL droplets per well in a 12-well plate. Invert the plate and incubate at 37℃ with 5% CO_2_ for 30 min to allow solidification.

v. Culture Maintenance: After matrix solidification, add 1 mL of complete ovarian cancer organoid medium (OCOM, preparation method in Table 4.4). Maintain at 37℃ with 5% CO_2_, replacing OCOM every 3 days.

2) PDOCO Passaging

i. Matrix Dissociation: Aspirate the medium and add pre-cooled DPBS to the well. Gently scrape the well to detach the BME-embedded PDOCOs, collect in a centrifuge tube, and pipette gently on ice to dissolve the matrix. Centrifuge at 300 ×g for 3 min.

ii. Enzymatic Dissociation: Discard the supernatant and resuspend in 200 µL TrypLE. Incubate at 37℃ for 3 min (monitor under a microscope; optimal digestion yields uniformly sized small cell clusters).

iii. Neutralization and Washing: Neutralize with 5× volume of AdDF12 and centrifuge at 240 ×g for 3 min.

iv. Re-embedding in BME Matrix: Wash once with AdDF12, centrifuge at 300 ×g, and remove all supernatant. Resuspend in BME matrix on ice, plate as droplets, and incubate inverted at 37℃ with 5% CO_2_ for 30 min.

v. Post-Passaging Culture: After matrix solidification, add OCOM and culture under standard conditions.

3) PDOCO Cryopreservation

The procedure follows the passaging steps until AdDF12 neutralization. After centrifugation: Discard the supernatant and resuspend in PDOCO freezing medium (AdDF12 with 80% FBS and 10% DMSO). Transfer to a controlled-rate freezing container and store at -80℃ before long-term preservation in liquid nitrogen.

4) PDOCO Thawing and Recovery

Retrieve the cryopreserved organoids from liquid nitrogen and immediately transfer them to a 37℃ water bath for rapid thawing. Once only small ice crystals remain, promptly neutralize the suspension with 5× volume of AdDF12. Centrifuge at 300 ×g for 3 min, discard the supernatant, and keep the pellet on ice. Resuspend the pellet in an appropriate volume of BME matrix, then plate as droplets. Invert the culture plate and incubate at 37℃ with 5% CO_2_ for 30 min to allow matrix solidification. Following solidification, add OCOM and maintain the culture at 37℃ with 5% CO_2_.

**Multiplex Immunofluorescence (MIF) Staining of PDOCOs**

Given that PDOCOs exist as three-dimensional spherical cell clusters, this study employed whole-mount staining combined with confocal laser scanning microscopy for analysis. The detailed protocol is as follows:

1) Organoid Collection: Harvest organoids and resuspend in DPBS in a 96-well plate.

2) Fixation: Under a stereomicroscope, carefully transfer appropriately sized organoids to tissue fixation solution using a capillary tube with mouth pipetting. Fix at room temperature for 30 min, then wash three times with PBS.

3) Permeabilization: Treat organoids with 0.2% Triton X-100 solution at room temperature for 20 min, followed by three PBS washes.

4) Blocking: Transfer organoids to 3% BSA-PBS solution and block at room temperature for 2 hours.

5) Primary Antibody Incubation: After PBS washing, incubate with diluted primary antibody at 4℃ overnight.

6) Secondary Antibody Staining: The following day, wash organoids three times with 0.05% Tween 20-PBS, then incubate with fluorescent secondary antibody at room temperature for 2 hours protected from light.

7) Nuclear Staining: Following PBS washes, transfer organoids to wells containing DAPI solution for nuclear staining.

8) Mounting and Imaging: After three final PBS washes, carefully transfer organoids to a confocal dish containing antifade mounting medium using mouth pipetting and capillary tubes. Proceed with confocal microscopy imaging or Z-stack acquisition.

**Molecular Docking Prediction**

The protein structure files of STING and SURF4, downloaded from AlphaFold (https://alphafold.ebi.ac.uk/), were uploaded to the rigid docking software GRAMM (https://gramm.compbio.ku.edu). The docking results were then imported into PDBePISA (https://www.ebi.ac.uk/pdbe/pisa/) to further analyze the binding interface and binding free energy, followed by visualization of the docking outcomes.

**Supplementary figure legends:**

**Fig. S1: NAMPT-mediated NAD^+^ biosynthesis regulates T cell function in OC microenvironment**

**A.** Functional enrichment analysis of gene clusters related to the regulation of immune responses in the OC microenvironment. **B.** Expression levels of NAD^+^ synthesis and metabolism-related genes across three immune subtypes, with NAMPT expression significantly lower in the immune-silent subtype compared to the other two subtypes. **C.** Dimensionality reduction and clustering of OC single-cell data, visualized based on sample origin. **D.** Discrimination between malignant and non-malignant OC cells based on WT1, PAX8, and MUC16 expression. **E.** Differential expression of NAD^+^ synthesis and metabolism-related genes in malignant versus non-malignant OC cells. **F.** Differential expression of NAD^+^ synthesis-related genes across various cell types. **G.** Positive correlation between the NAD^+^ metabolism-related gene NAMPT and the expression of anti-tumor effector genes in microenvironmental T cells. **H.** Analysis of signaling pathways related to the functional regulation of NAMPT in T cells.

**Fig. S2: NAMPT and exogenous NAM supplementation enhance T cell** **proliferation and chemotaxis**

**A.** Overexpression of NAMPT promotes T cell proliferation, while inhibition of the STING axis suppresses the promoting effect of NAMPT on T cell proliferation. **B-C.** Overexpression of NAMPT increases the number of T cells in the S/G2M phase. **D.** PMA and ionomycin can successfully induce T cell activation in vitro. **E.** The regulatory effects of NAD^+^ precursors NAM, NMN, and NR on OC cell proliferation, with NMN promoting the proliferation of OC cells. **F-G.** Reducing NAD^+^ levels in T cells inhibits their proliferation, whereas supplementing with NAM to increase NAD^+^ levels promotes T cell proliferation. **H-I.** Lowering NAD^+^ levels in T cells decreases the number of T cells in the S/G2M phase, while supplementing with NAM to increase NAD^+^ levels raises the number of T cells in the S/G2M phase. **J.** Supplementing with NAM to increase NAD^+^ levels upregulates the expression of T cell chemotactic receptors.

**Fig. S3: Exogenous NAM supplementation enhances T cell-mediated anti-tumor activity**

**A-C.** The expression of T cell anti-tumor activation proteins is positively correlated with T cell NAD^+^ levels. **D.** Supplementing with NAM increases the secretion of GZMB and IFNγ by T cells. **E.** Preliminary cytotoxicity assays revealed that the killing effect increased significantly at an effector-to-target (E:T) ratio of 9:1. Although further elevation of the E:T ratio resulted in enhanced cytotoxicity, no statistically significant difference was observed. **F-G.** NAM enhances the cytotoxic ability of T cells. **H.** NAM enhances the inhibitory effect of T cells on the proliferation of HEY cells. **I-J.** The NAD^+^ level in T cells influences their cytotoxic effect on SKOV3, with higher NAD^+^ levels promoting T cell-induced apoptosis of SKOV3. **K.** NAM supplementation alleviates OC cell-induced exhaustion in T cells. **L-M.** The NAD^+^ level in T cells is negatively correlated with their exhaustion state.

**Fig. S4: The p-STING/p-IRF3 axis plays a crucial role in NAD^+^-mediated activation of T cell function and anti-tumor immunity**

**A-B.** The activity of the p-STING/p-IRF3 axis in T cells is positively correlated with T cell NAD^+^ levels, and increasing NAD^+^ levels can activate the p-STING axis in T cells. **C.** The cGAS inhibitor RU.521 has a weak inhibitory effect on NAM-induced activation of T cell anti-tumor effects, while the STING inhibitor H-151 significantly inhibits NAM-mediated activation of T cell anti-tumor function. **D.** After STING inhibition, the expression of NAM-induced anti-tumor factors in T cells is suppressed. **E.** After STING inhibition in T cells, the NAM-enhanced cytotoxic effect of T cells on OC cells is suppressed, and the inhibitory effect on OC cell proliferation is weakened. **F.** After STING inhibition, the NAM-upregulated expression of T cell chemotaxis receptors decreases. **G-J.** Inhibition of the STING axis weakens the NAM-promoted proliferation of T cells, significantly reducing the number of cells in the S/G2M phase.

**Fig. S5: Elevated NAD^+^ levels promote ubiquitination-mediated degradation of SURF4**

**A.** The protein-protein docking prediction analysis demonstrated an effective binding interaction between STING and SURF4 proteins. **B.** SURF4 and STING interact within T cells. **C.** SURF4 and STING interact in HEK 293T cells. **D.** Transcriptional level detection of SURF4 knockdown in T cells. **E.** Overexpression of NAMPT does not affect the transcriptional level of SURF4 in T cells. **F.** Fluctuations in NAD^+^ levels do not affect the transcriptional level of SURF4 in T cells. **G.** Bioinformatics analysis predicts that the SURF4 protein undergoes ubiquitination modification. **H-I.** Bioinformatics analysis predicts potential associations between the ubiquitin E3 ligase PELI1/deubiquitinase USP47 and both NAMPT and SURF4. **J-K.** NAM-mediated NAD^+^ elevation downregulates USP47 expression in T cells. **L.** Intracellular USP47 levels show a negative correlation with the expression of multiple antitumor genes in T cells.

**Fig. S6: Enhancing T cell NAD^+^ levels potentiates anti-tumor immunity during PARPi treatment in OC**

**A.** PARPi Olaparib has no direct effect on the STING axis and downstream anti-tumor signaling in T cells. **B.** PARPi-treated OC cells significantly activate the STING axis and anti-tumor activity in T cells. **C-D.** The activation of T cell anti-tumor function by PARPi-treated OC cells is influenced by T cell NAD^+^ levels. When NAD^+^ levels are low, the activation of T cells by PARPi-treated OC cells is suppressed. **E.** PARPi-treated OC cells promote the aggregation of STING on the Golgi apparatus in T cells, making the stability of STING on the Golgi apparatus more sensitive to fluctuations in NAD^+^ levels. **F-G.** The combination of NAM and Olaparib significantly enhances the inhibitory effect of T cells on the proliferation of PDOCO.

**Supplementary table legends:**

**Table S1.** The basic information of clinical samples used to construct PDOCO in this study.

**Table S2.** Formulation of PDOCO Culture Medium.

**Table S3.** The primer sequences for all genes used in RT-qPCR experiments in this study.

**Table S4.** Detailed parameters of the STING-SURF4 protein docking prediction model.

**References**

1. Hippen AA, Omran DK, Weber LM, Jung E, Drapkin R, Doherty JA*, et al.* Performance of computational algorithms to deconvolve heterogeneous bulk ovarian tumor tissue depends on experimental factors. *Genome Biol* 2023, **24**(1)**:** 239.

2. Ivich A, Davidson NR, Grieshober L, Li W, Hicks SC, Doherty JA*, et al.* Missing cell types in single-cell references impact deconvolution of bulk data but are detectable. *Genome Biol* 2025, **26**(1)**:** 86.
