# Supplementary figures and images for: NAD+ Metabolism-Mediated SURF4-STING Axis Enhances T-Cell Anti-Tumor Effects in the Ovarian Cancer Microenvironment
Source: Cell Death Dis. 2025 Aug 23;16(1):640. doi: 10.1038/s41419-025-07939-9 (PMC12373823; doi:10.1038/s41419-025-07939-9)

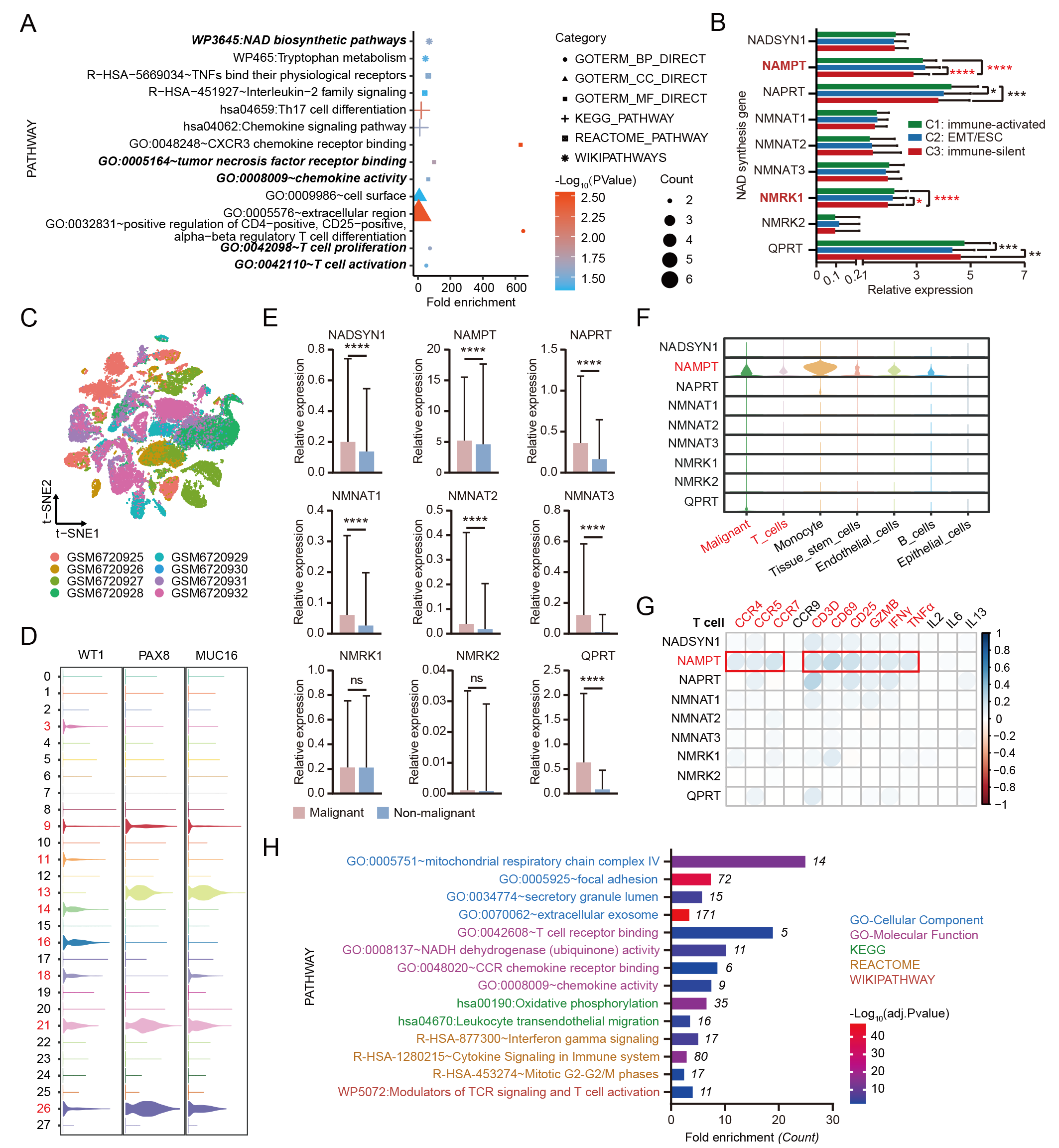

Supplement: Supplementary file 2 — Supplementary Figure 1 [file 41419_2025_7939_MOESM2_ESM.tif]

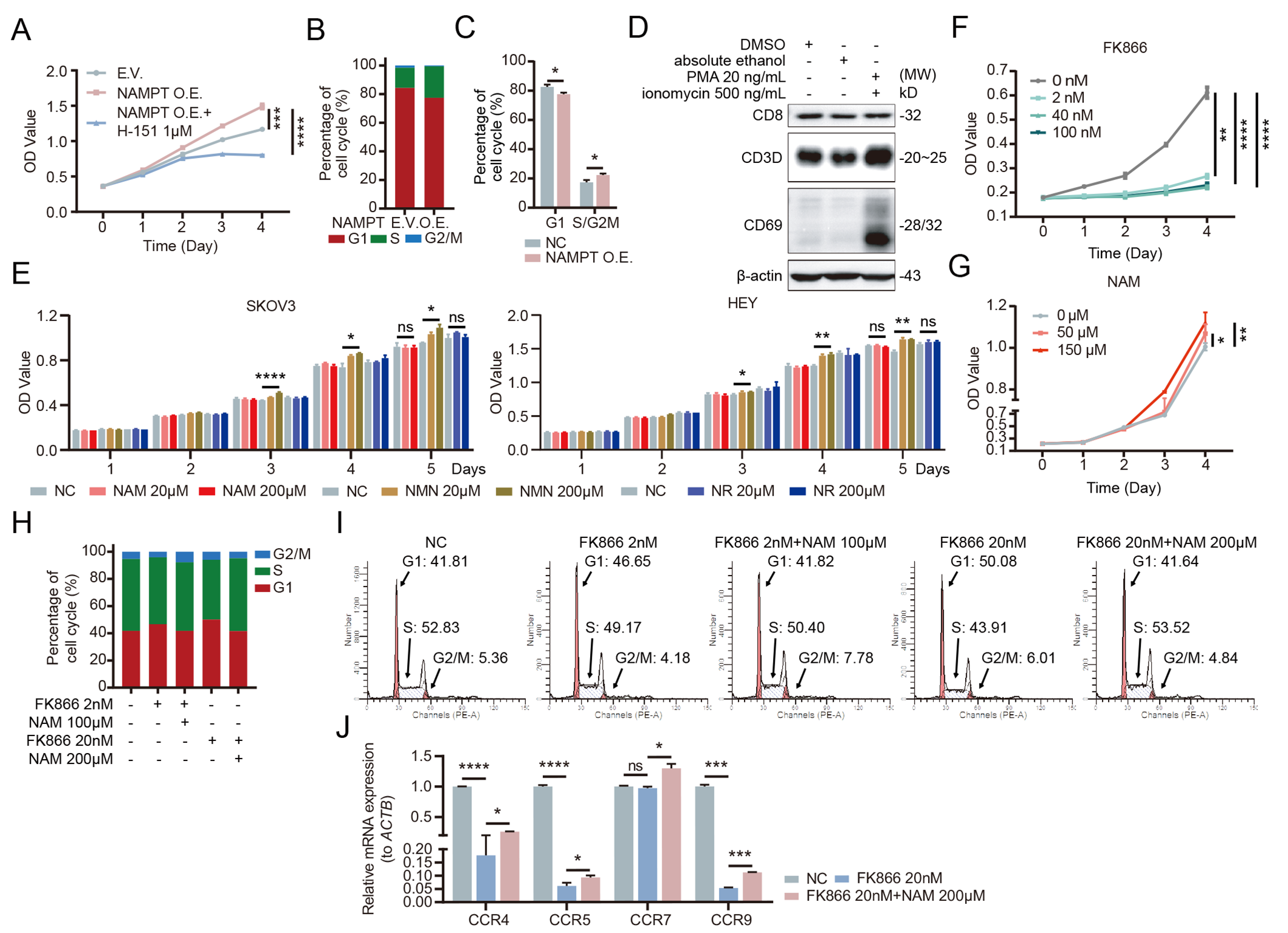

Supplement: Supplementary file 3 — Supplementary Figure 2 [file 41419_2025_7939_MOESM3_ESM.tif]

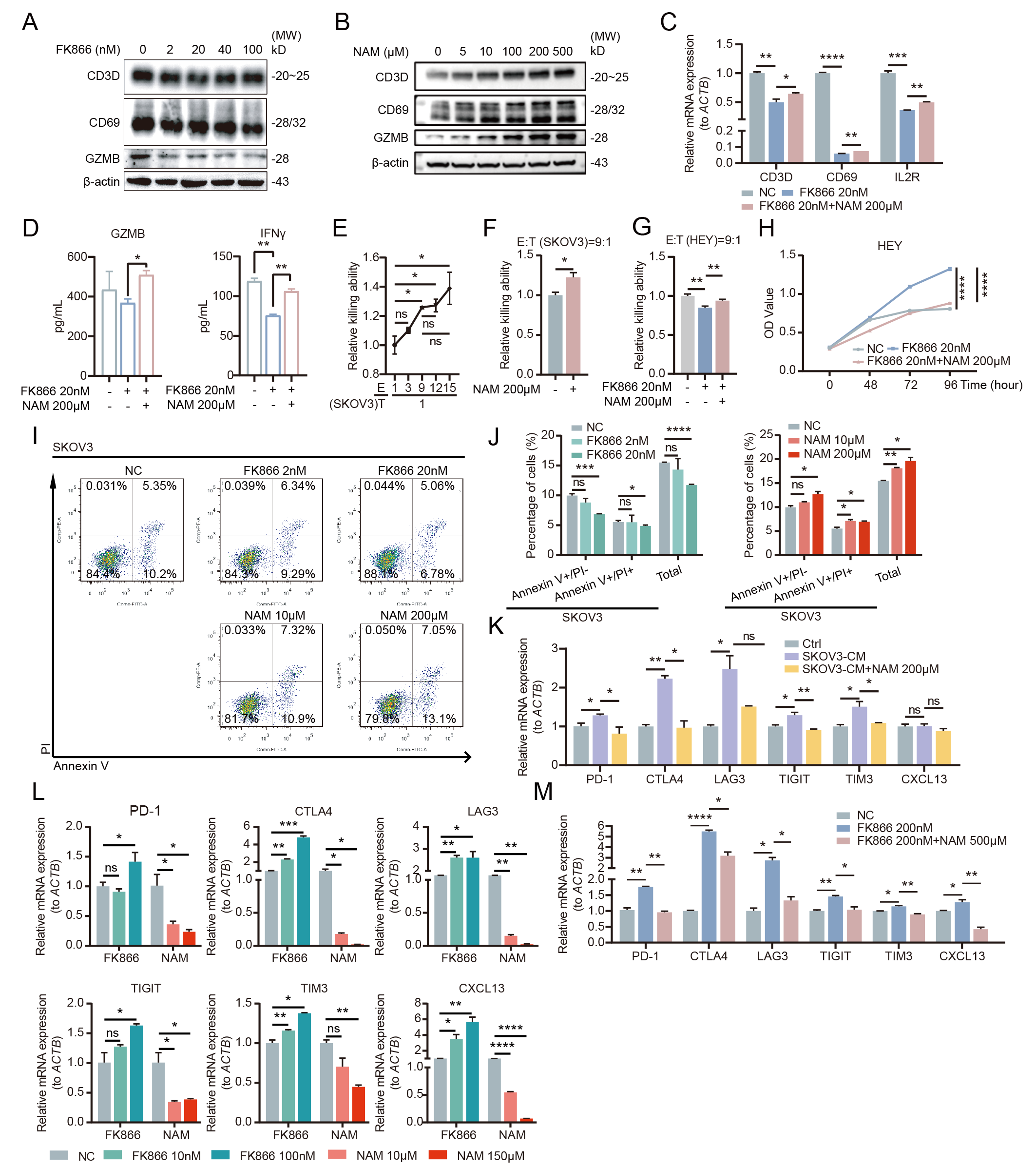

Supplement: Supplementary file 4 — Supplementary Figure 3 [file 41419_2025_7939_MOESM4_ESM.tif]

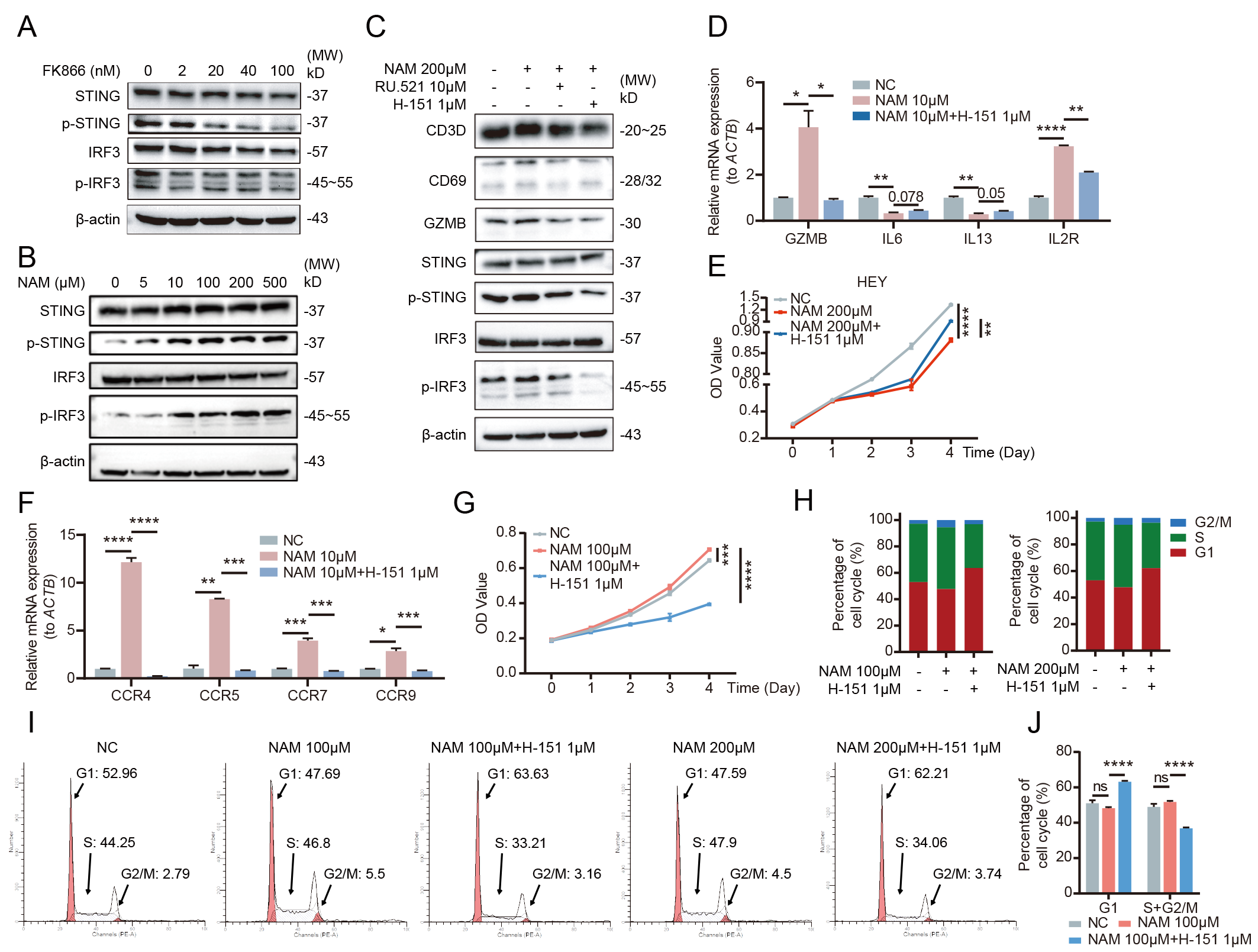

Supplement: Supplementary file 5 — Supplementary Figure 4 [file 41419_2025_7939_MOESM5_ESM.tif]

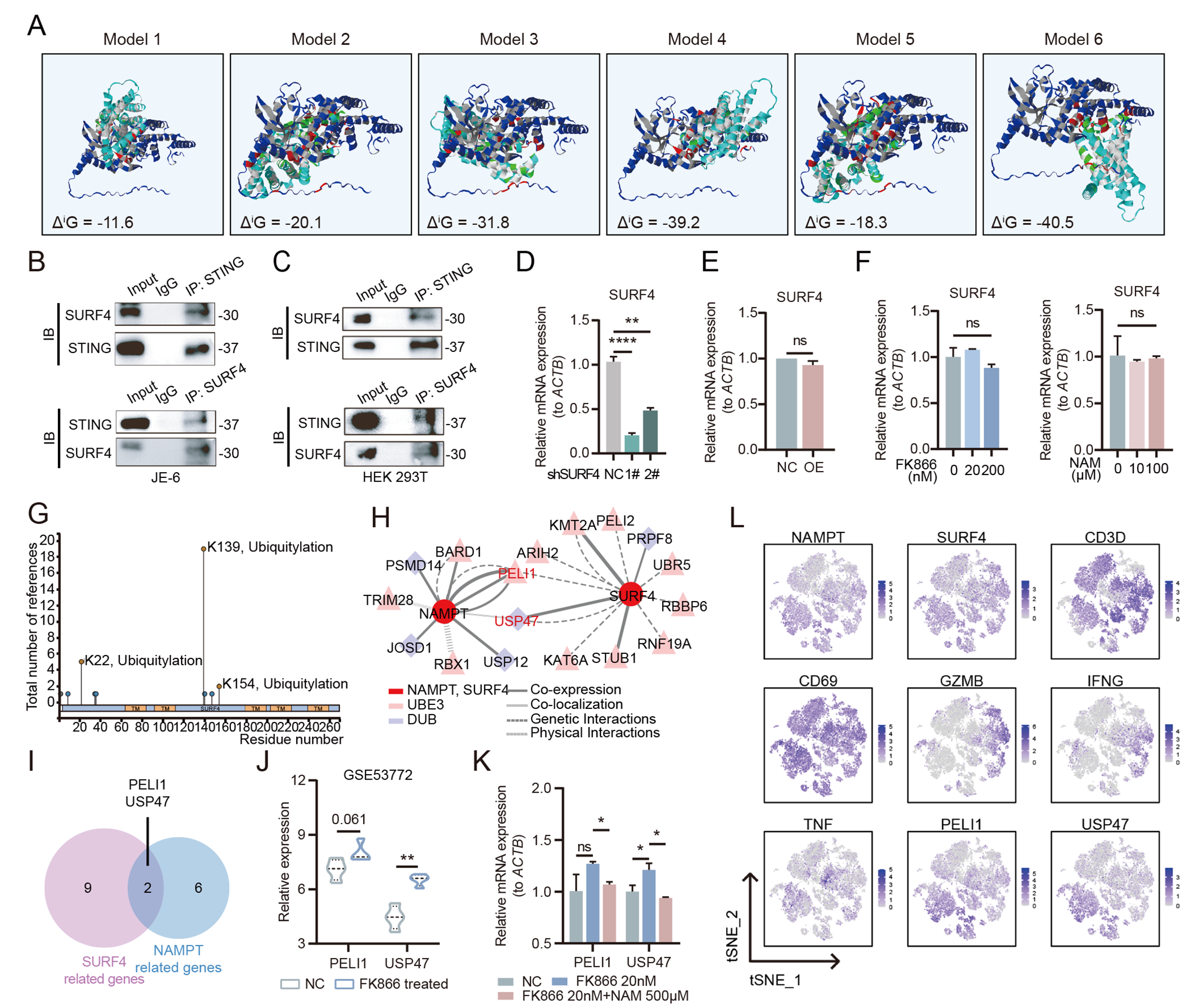

Supplement: Supplementary file 6 — Supplementary Figure 5 [file 41419_2025_7939_MOESM6_ESM.tif]

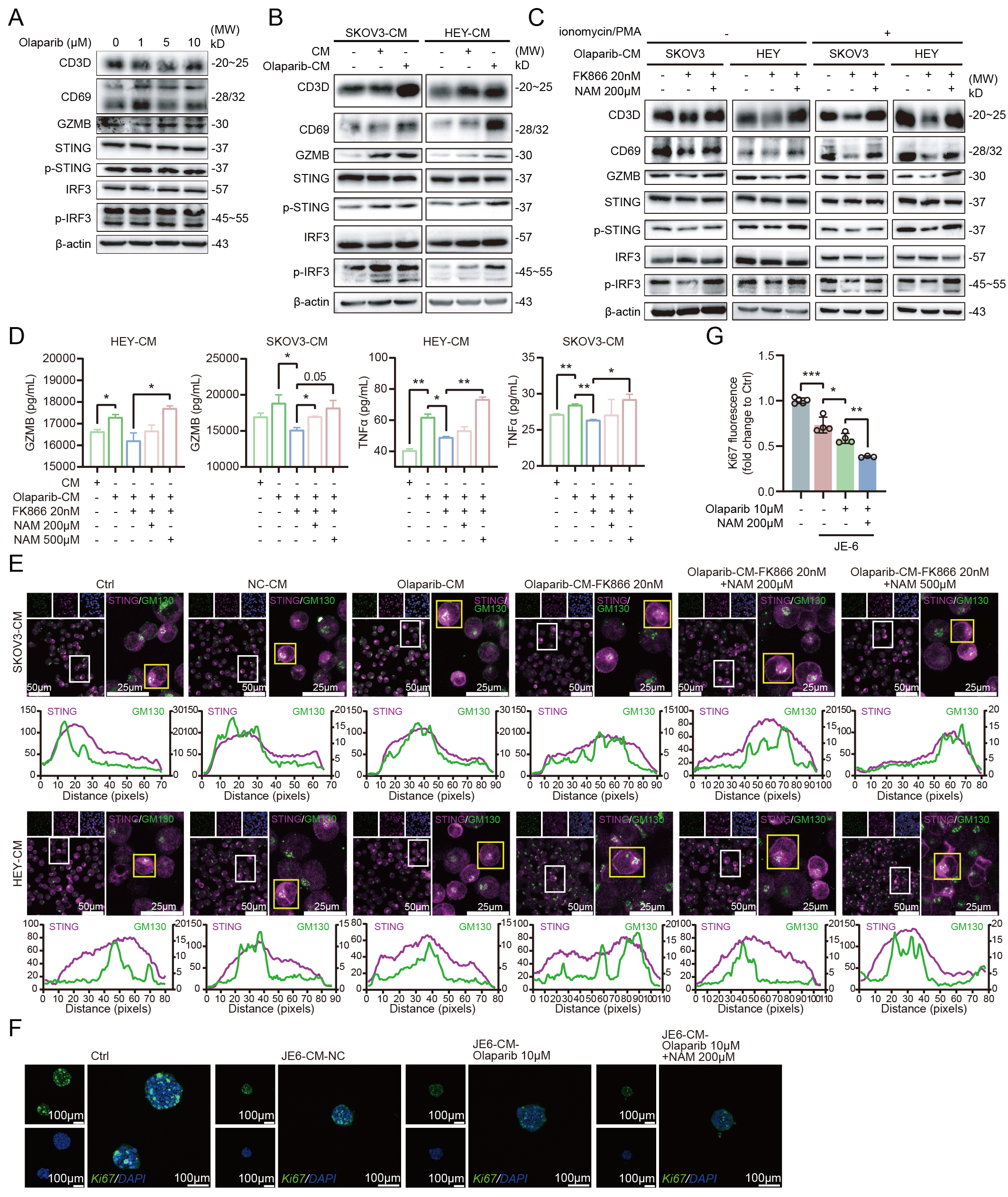

Supplement: Supplementary file 7 — Supplementary Figure 6 [file 41419_2025_7939_MOESM7_ESM.tif]
