# Supplementary material for: NAD+ Metabolism-Mediated SURF4-STING Axis Enhances T-Cell Anti-Tumor Effects in the Ovarian Cancer Microenvironment
Source: Cell Death Dis. 2025 Aug 23;16(1):640. doi: 10.1038/s41419-025-07939-9 (PMC12373823; doi:10.1038/s41419-025-07939-9)
Supplement: Supplementary file 12 — Original Western Blot Images [file 41419_2025_7939_MOESM12_ESM.pdf]

Fig. 1L, Fig. 3A

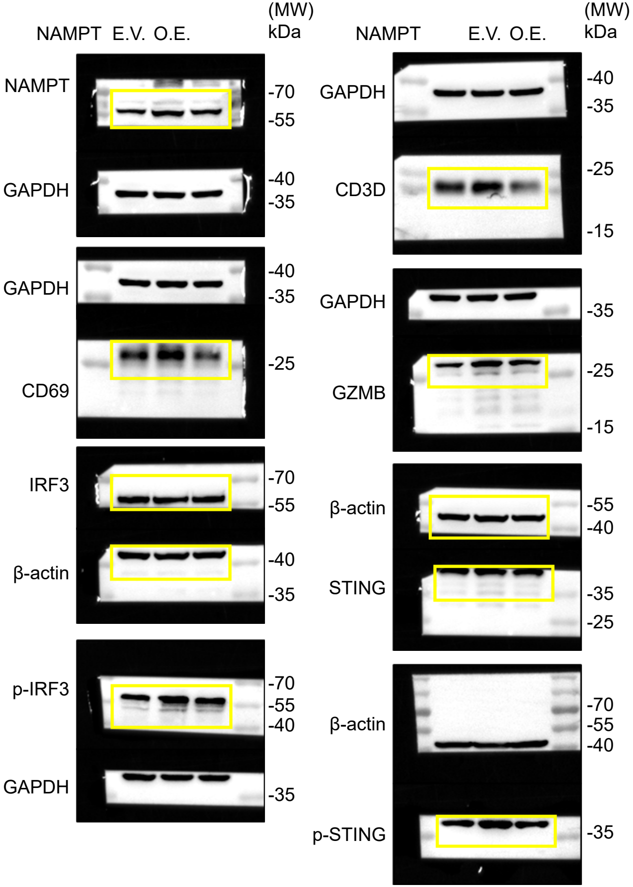

Fig. 2E, Fig. 3C

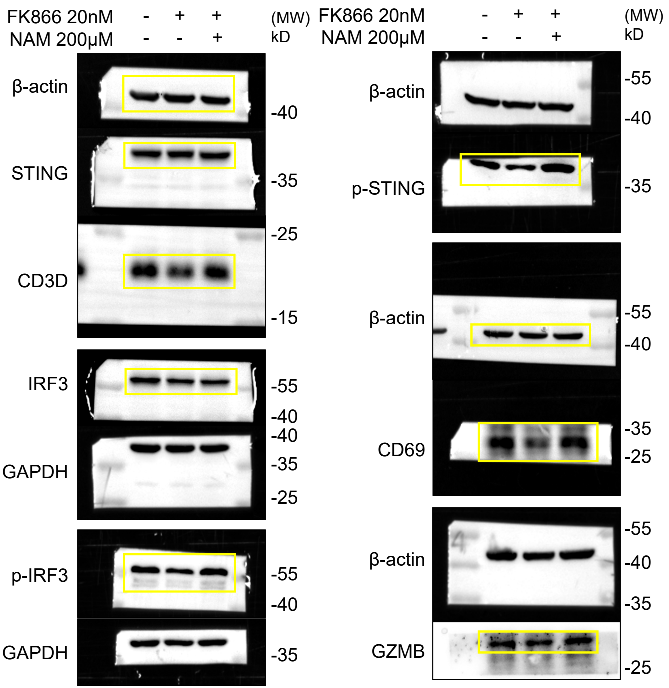

Fig. 3E, G

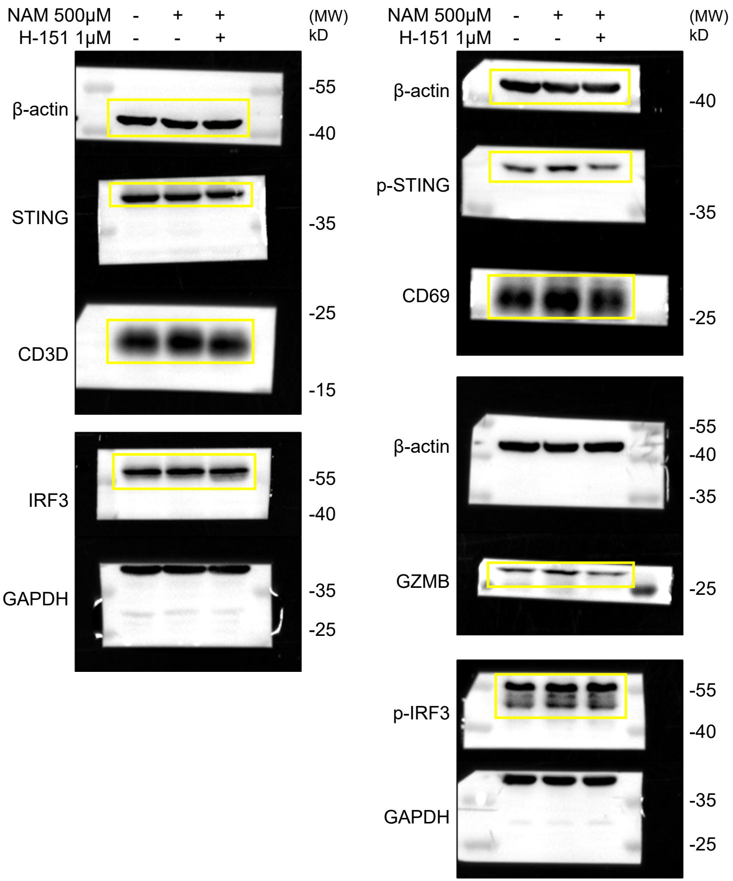
NAM 500μM - + +
H-151 1μM - - +
(MW) kD
β-actin
STING
CD3D
IRF3
GAPDH
-55
-40
-35
-25
-15
-55
-40
-35
-25
NAM 500μM - + +
H-151 1μM - - +
(MW) kD
β-actin
p-STING
CD69
-40
-35
-25
β-actin
GZMB
-55
-40
-35
-25
p-IRF3
GAPDH
-55
-40
-35
-25

Fig. 4F

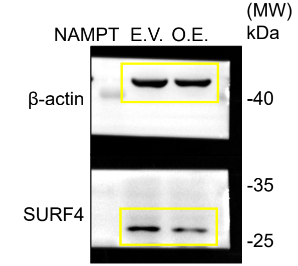

Fig. 4H

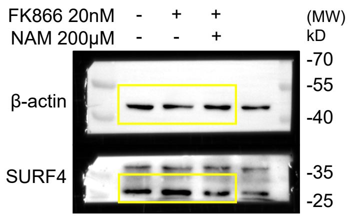

Fig. 4K

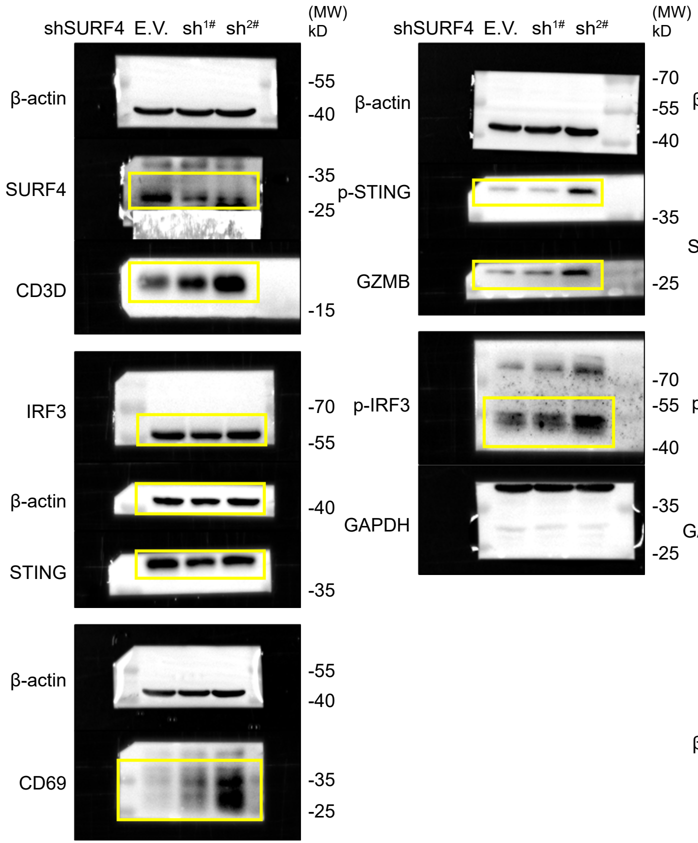

Fig. 4L

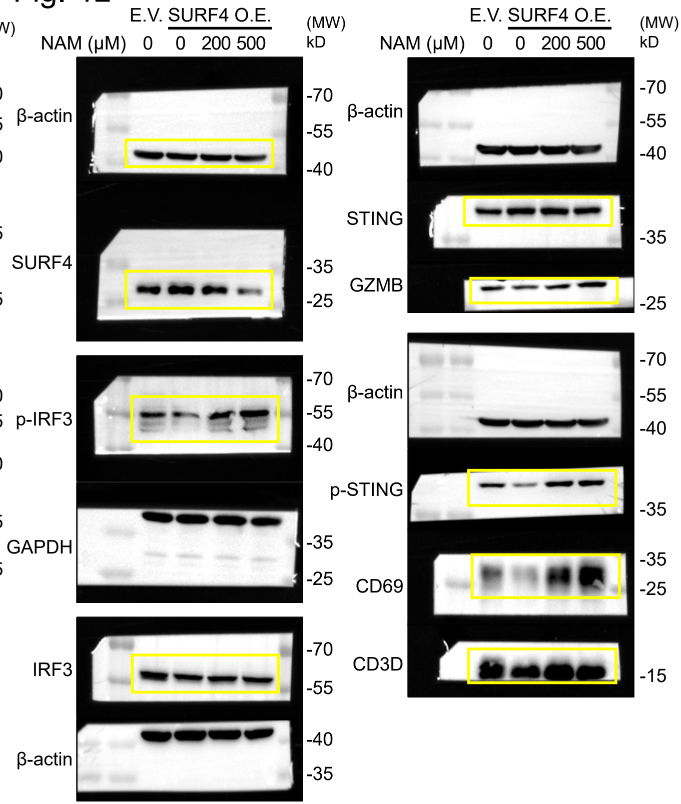

Fig. 4M

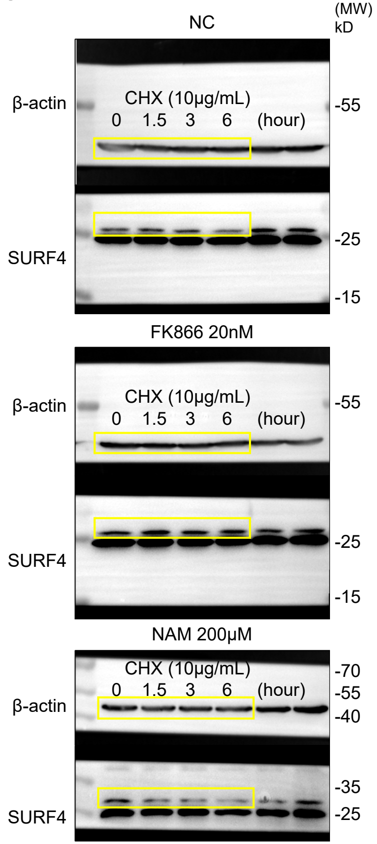

Fig. 4Q

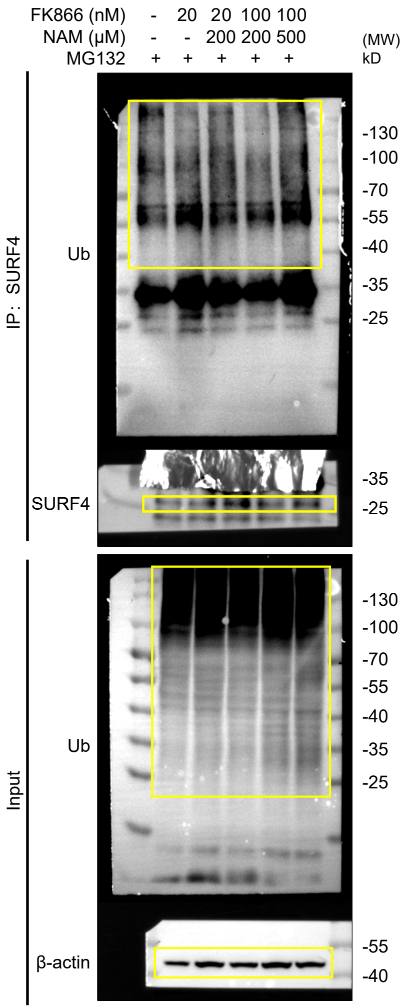

Fig. 4R

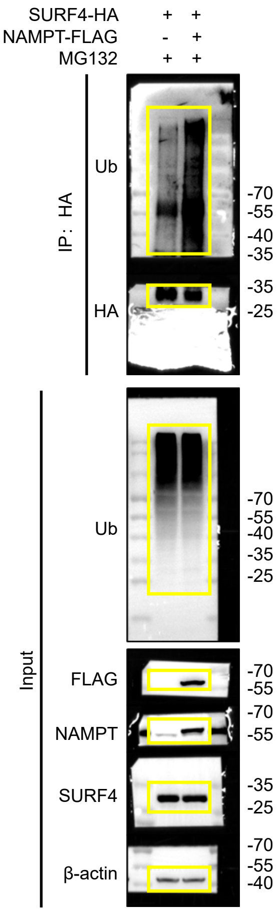

Fig. 4O

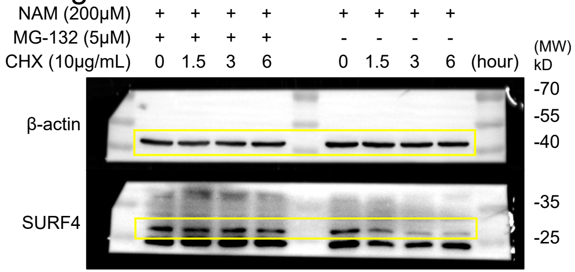

Fig. 5C

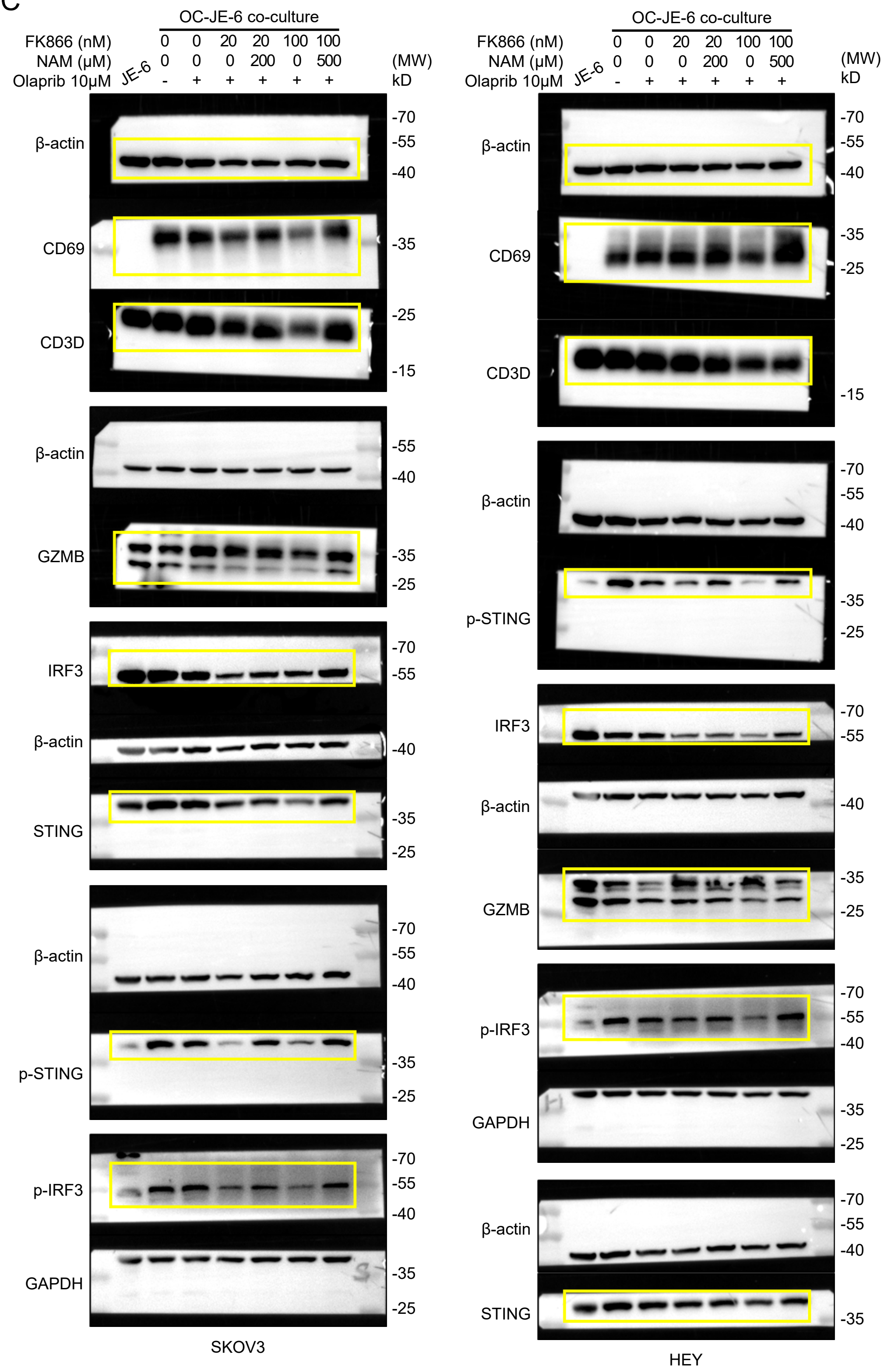
OC-JE-6 co-culture
FK866 (nM) 0 0 20 20 100 100
NAM (μM) 0 0 0 200 0 500
Olaprib 10μM JE-6 - + + + + +
(MW) kD
β-actin
CD69
CD3D
GZMB
IRF3
STING
p-STING
p-IRF3
GAPDH
-70
-55
-40
-35
-25
-15
SKOV3
HEY

Fig. S2D

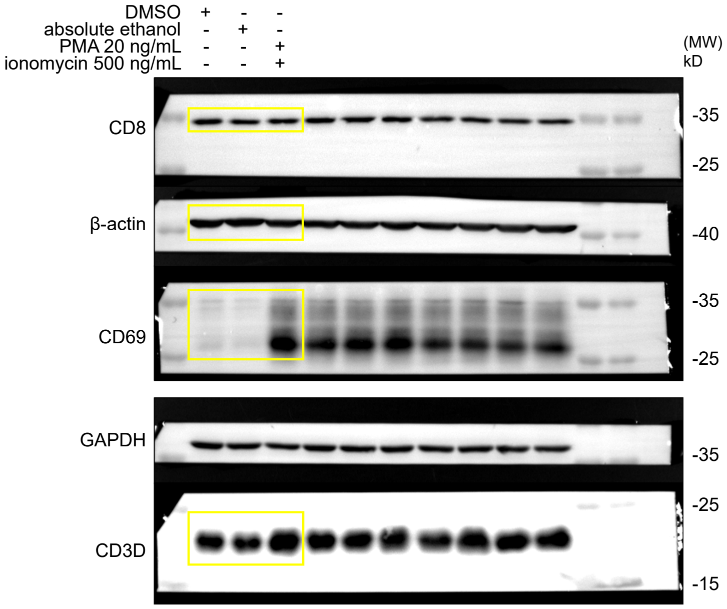

Fig. S3A, S4A

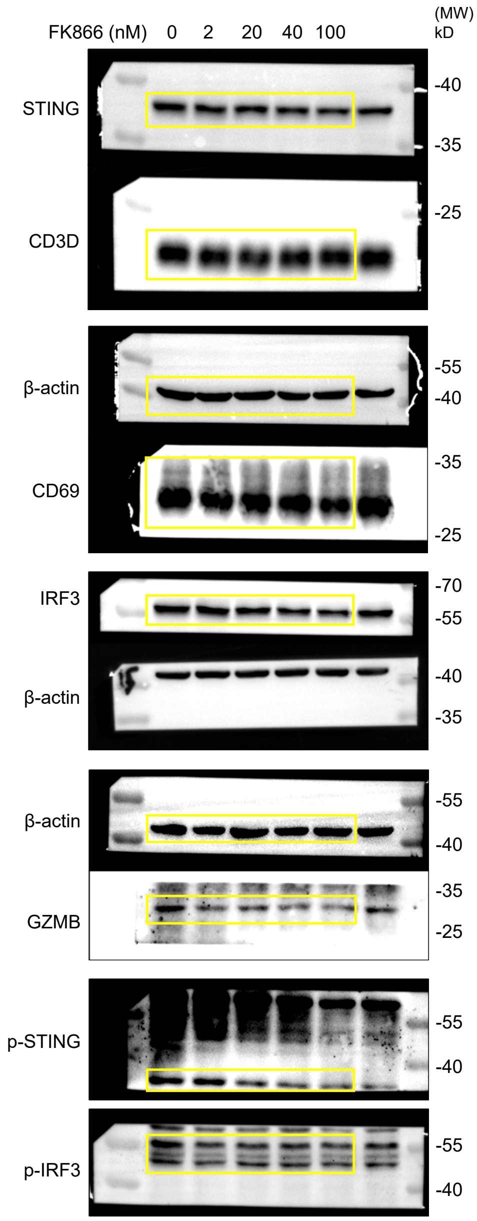

Fig. S3B, S4B

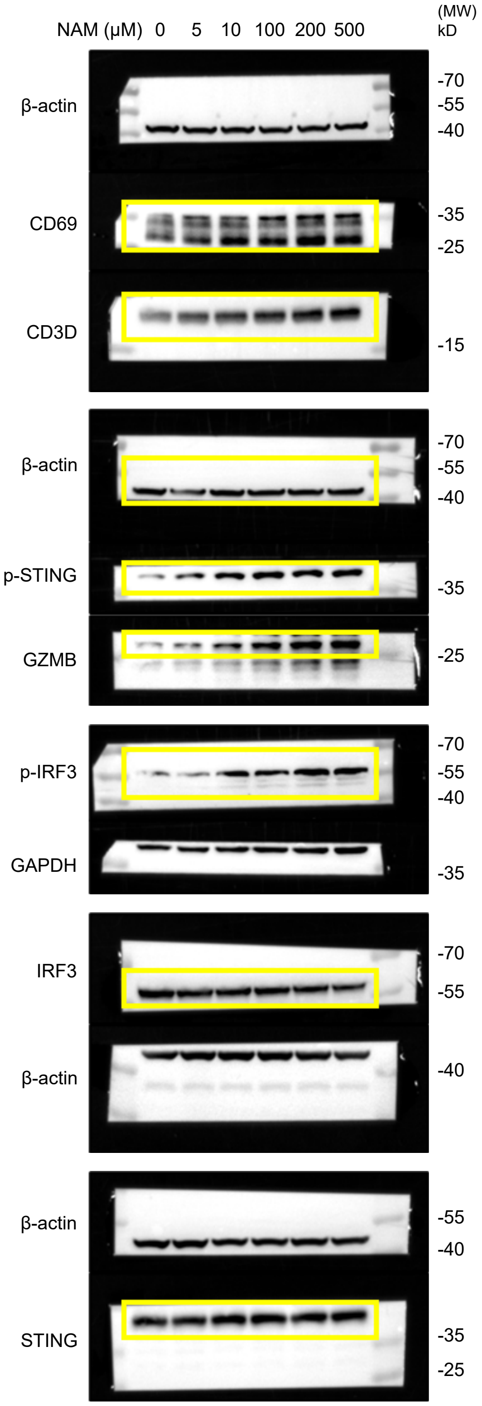

Fig. S4C

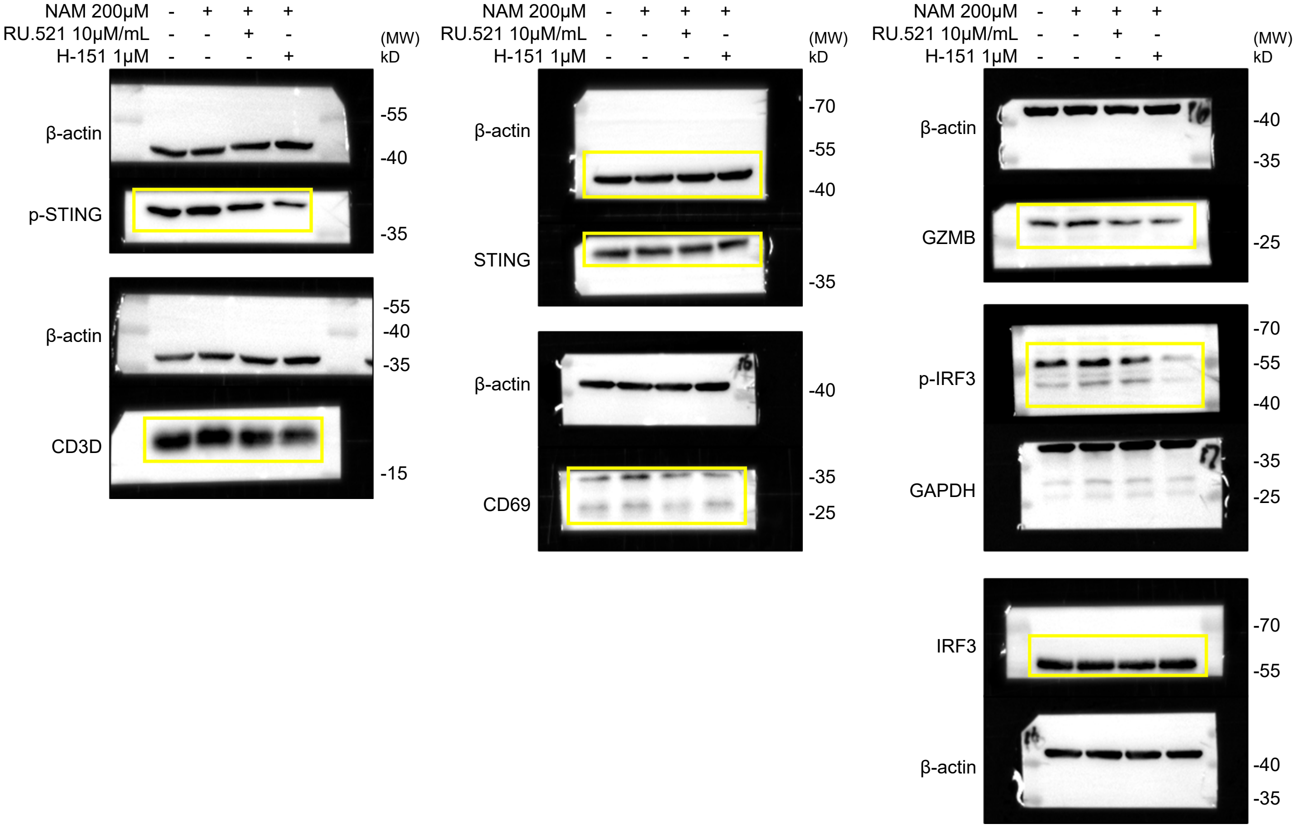
NAM 200μM - + + +
RU.521 10μM/mL - - + -
H-151 1μM - - - +
(MW) kD
β-actin
p-STING
β-actin
CD3D
-55
-40
-35
-55
-40
-35
-15
NAM 200μM - + + +
RU.521 10μM/mL - - + -
H-151 1μM - - - +
(MW) kD
β-actin
STING
β-actin
CD69
-70
-55
-40
-35
-40
-35
-25
NAM 200μM - + + +
RU.521 10μM/mL - - + -
H-151 1μM - - - +
(MW) kD
β-actin
GZMB
p-IRF3
GAPDH
IRF3
β-actin
-40
-35
-25
-70
-55
-40
-35
-25
-70
-55
-40
-35

Fig. S5B

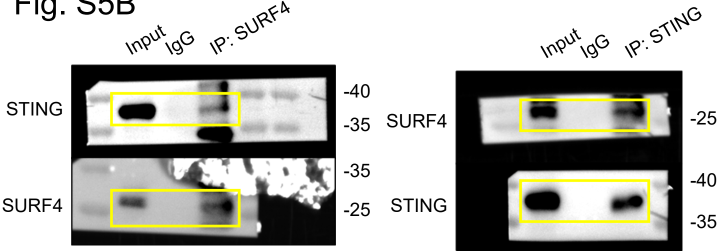

Fig. S5C

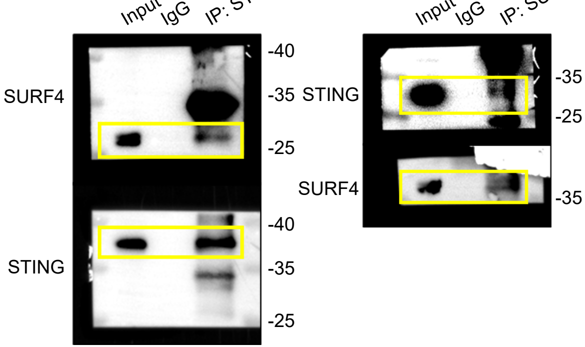

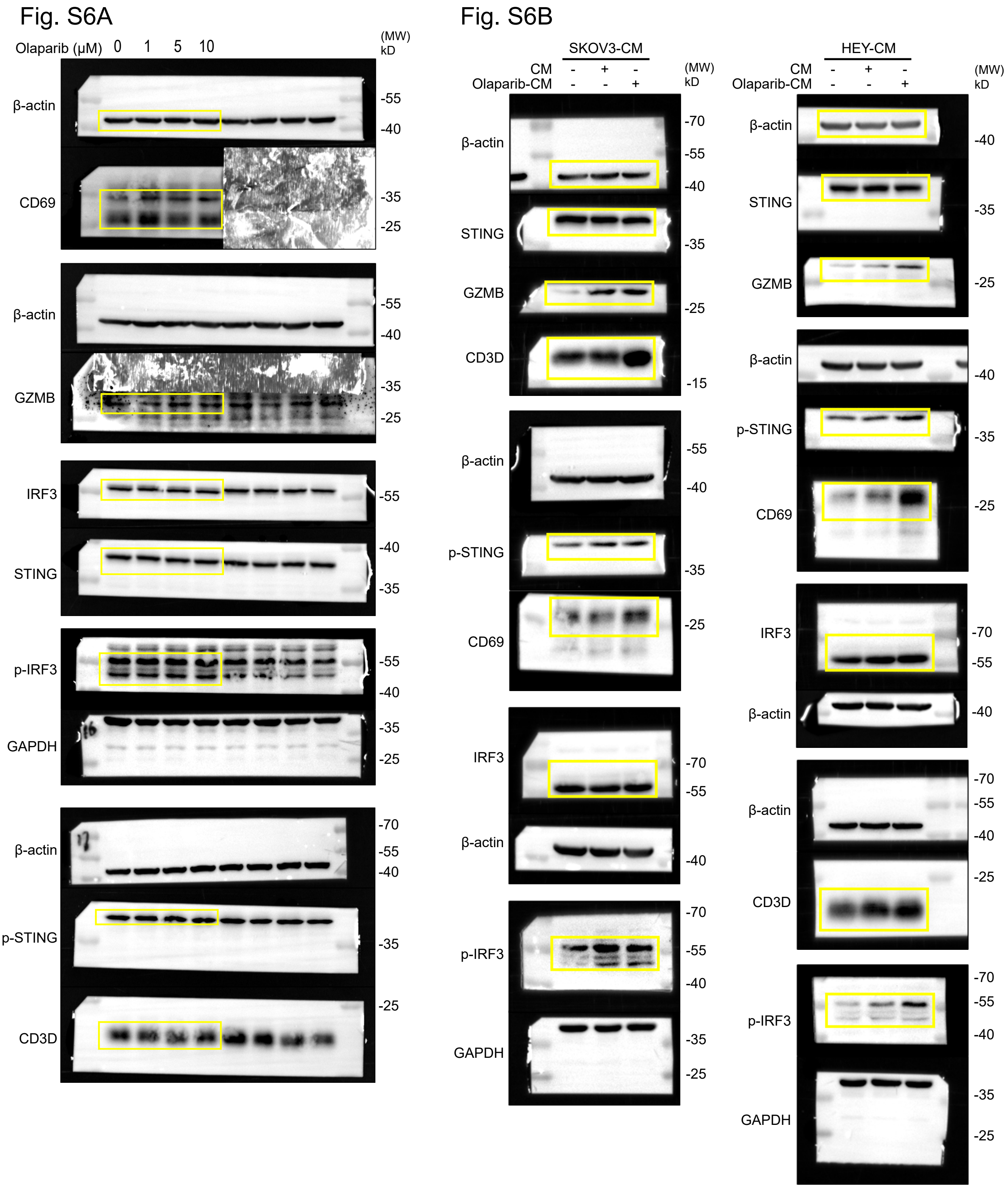
Fig. S6A
Olaparib (μM) 0 1 5 10
(MW) kD
β-actin
CD69
GZMB
IRF3
STING
p-IRF3
GAPDH
p-STING
CD3D
-55
-40
-35
-25
-70
Fig. S6B
SKOV3-CM
HEY-CM
CM - + -
Olaparib-CM - - +
-15

Fig. S6C

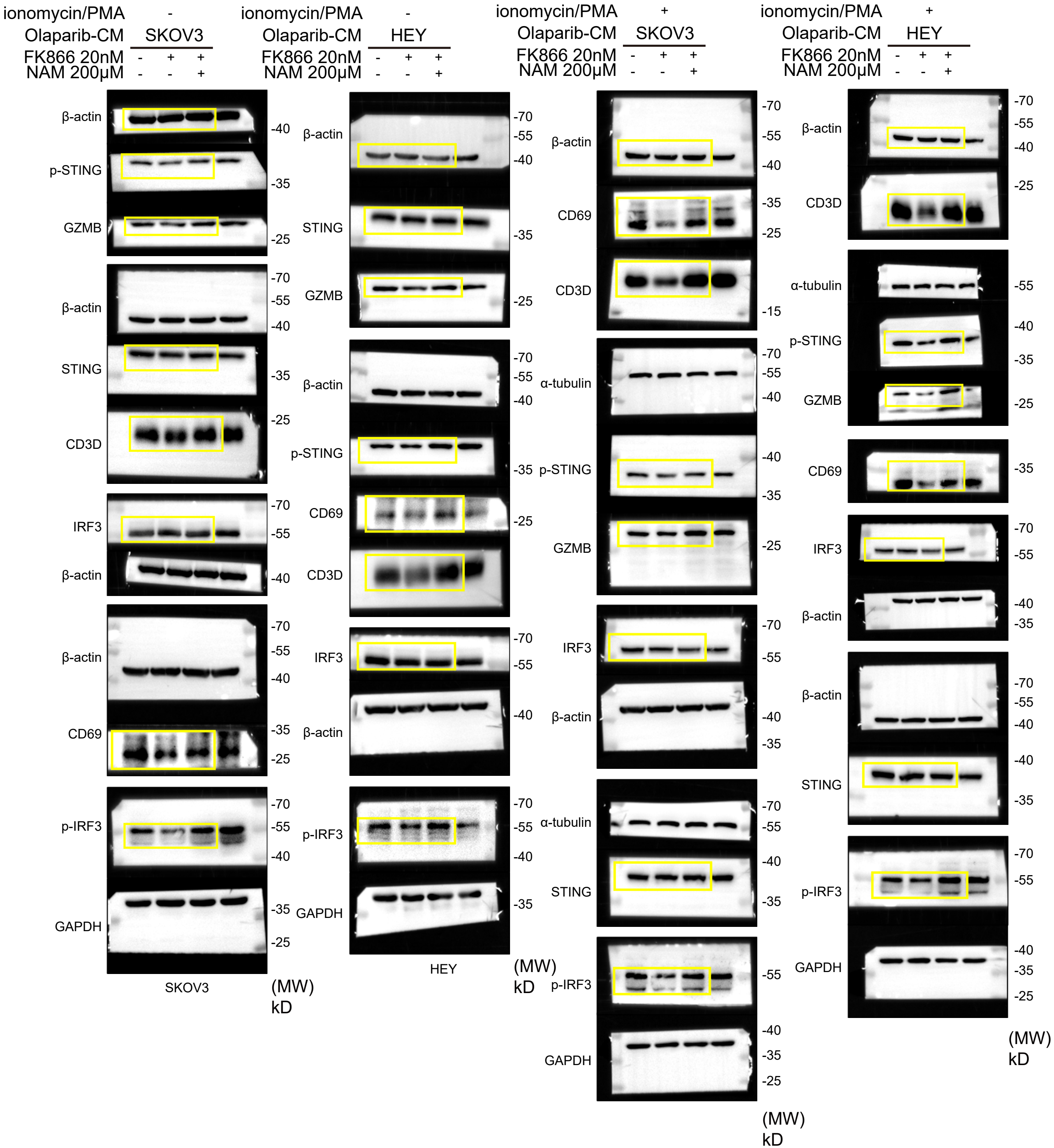
ionomycin/PMA -
Olaparib-CM SKOV3
FK866 20nM - + +
NAM 200μM - - +
ionomycin/PMA -
Olaparib-CM HEY
FK866 20nM - + +
NAM 200μM - - +
ionomycin/PMA +
Olaparib-CM SKOV3
FK866 20nM - + +
NAM 200μM - - +
ionomycin/PMA +
Olaparib-CM HEY
FK866 20nM - + +
NAM 200μM - - +
β-actin
p-STING
GZMB
STING
CD3D
IRF3
CD69
p-IRF3
GAPDH
α-tubulin
-70
-55
-40
-35
-25
-15
SKOV3
HEY
(MW)
kD
